# Gender and residential differences in sleep quality among Chinese adolescents aged 13–18 years

**DOI:** 10.1371/journal.pone.0349681

**Published:** 2026-06-10

**Authors:** Xuye Kang, Jianying Li, Huipan Wu

**Affiliations:** 1 School of Physical Education, Shanxi University, Taiyuan, China; 2 School of Physical Education, Taiyuan Institute of Technology, Taiyuan, China; University of Rijeka Faculty of Health Studies: Sveuciliste u Rijeci Fakultet zdravstvenih studija, CROATIA

## Abstract

**Background:**

Good sleep is essential for healthy adolescent development, yet declining sleep quality among Chinese adolescents has become a nationwide public health concern. Residential setting and sex are key determinants of sleep quality; however, systematic evaluations of sleep quality across regions and between sexes among Chinese adolescents remain limited.

**Methods:**

Data were collected on Pittsburgh Sleep Quality Index (PSQI), body mass index (BMI), Physical Fitness Index (PFI), sedentary time, screen time, and mental health from 5,713 adolescents (mean age, 15.11 ± 1.70 years). Logistic regression models were applied to examine the effects of these factors on adolescent sleep quality, including interactions with residence and gender.

**Results:**

Overall, 33.71% of Chinese adolescents exhibited poor sleep quality. The prevalence of poor sleep quality was 35.78% among rural adolescents, which was significantly higher than that among urban adolescents (p < 0.001). Among females, 38.40% were classified as having poor sleep quality, a proportion significantly higher than that among males (p < 0.001). BMI, PFI, moderate-to-vigorous physical activity (MVPA), sedentary time, screen time, and mental health scores were significantly effects on the PSQI global score and its component scores (p < 0.05). Residence and gender significantly moderated the linear associations between sleep quality and BMI, PFI, MVPA, sedentary time, and mental health scores (p < 0.05).

**Conclusions:**

Chinese adolescents living in rural areas and female adolescents experience a greater burden of poor sleep quality. Mental health and PFI appear to be protective factors for sleep quality, whereas BMI, sedentary time, and screen time are risk factors among Chinese adolescents. Moreover, these associations are moderated by residence and gender.

## Introduction

Sleep occupies approximately one-third of the human lifespan, its quality is paramount for maintaining normal brain and bodily physiological functions and is increasingly recognized as a critical lifestyle determinant of physical and mental health. Sleep quality serves as a pivotal indicator of sleep status, comprising quantitative metrics-such as sleep duration, sleep latency, and the frequency of awakenings-as well as qualitative dimensions, including the subjective perception of sleep status and the sense of restoration post-awakening [1]. Although the direct benefits of high-quality sleep have yet to be fully quantified across diverse populations, sleep insufficiency or insomnia is universally acknowledged as a severe public health challenge. With the accelerating pace of modern life and escalating competitive pressures, compromised sleep quality has emerged as a significant issue afflicting the adolescent population. Research indicates that approximately 80% of Chinese adolescents average only 7.7 hours of sleep per night, falling short of the recommended healthy range of 8–10 hours [2,3]. The physiological development of adolescents is particularly susceptible to sleep disturbances, manifesting as impaired neurocognitive function, diminished well-being, and an elevated risks of depression, obesity, cardiovascular disease, and even unnatural death [4–10].

A substantial body of epidemiological research indicates that variations in geographic, social, demographic, and cultural factors may influence sleep quality and quantity at the population level [11–15]. Jenner et al. reported that the evolution of environmental and social factors associated with sleep settings shapes sleep patterns within a given society, therefore, findings from sleep-quality research conducted in affluent urban areas may not be generalizable to rural areas with different environmental contexts [16]. To date, numerous studies have conducted context-specific investigations of sleep quality in political, economic, and cultural centers such as Beijing and Shanghai, as well as in surrounding urban and rural areas, underscoring the importance of place of residence as a determinant of sleep quality [17,18]. However, in China, the household registration (hukou) system has, to some extent, constrained population mobility over recent decades, limiting the applicability of these findings to residents in other regions [19]. Meanwhile, reliable estimates of sleep quality among Chinese residents remain scarce, resulting in limited scholarly understanding of sleep quality disparities across different residence.

Given gender-specific variations in lifestyle, hormonal profiles, and sociocultural norms, sleep-quality problems in adolescents may vary by gender [20]. A large body of evidence indicates that both male and female adolescents commonly experience insufficient sleep duration, difficulty initiating sleep, and insomnia, however, the challenges appear more pronounced among female adolescents [21,22]. Galland et al., in a study of adolescents aged 15–17, reported significantly poorer sleep quality in females than in males [23]. This pattern emerges after menarche and remains relatively stable throughout adolescence [24]. Regarding determinants of sleep quality, poor sleep-hygiene practices, escalating mental health issues, reduced time spent in physical activity, the widespread use of electronic devices, and increased consumption of caffeinated foods and beverages have all been associated, to varying degrees, with declining sleep quality in both male and female adolescents [23,25–29]. However, studies indicate substantial gender differences in the relative contributions of these factors to sleep disturbances, In particular, sleep quality among female adolescents appears more susceptible to mental health problems such as anxiety and depression [10,25]. In addition, compared with males, female adolescents tend to consume more caffeinated drinks and foods, especially after dinner [23,30].

This study aims to conduct a nationwide cross-sectional investigation of sleep quality among Chinese adolescents, comparing residence and gender differences, and examining how age, mental health scores, body mass index (BMI), time spent in moderate-to-vigorous physical activity (MVPA), Physical Fitness Index (PFI), sedentary time, and screen time are associated with sleep quality across residential location and between gender. The primary hypotheses are that: (1) sleep quality would be poorer among urban than rural adolescents; (2) sleep quality would be poorer among females than males; and (3) residence (urban vs. rural) and gender would moderate the effects of these factors and adolescent sleep quality.

## Materials and methods

### Research design and participants

**Fig 1:**
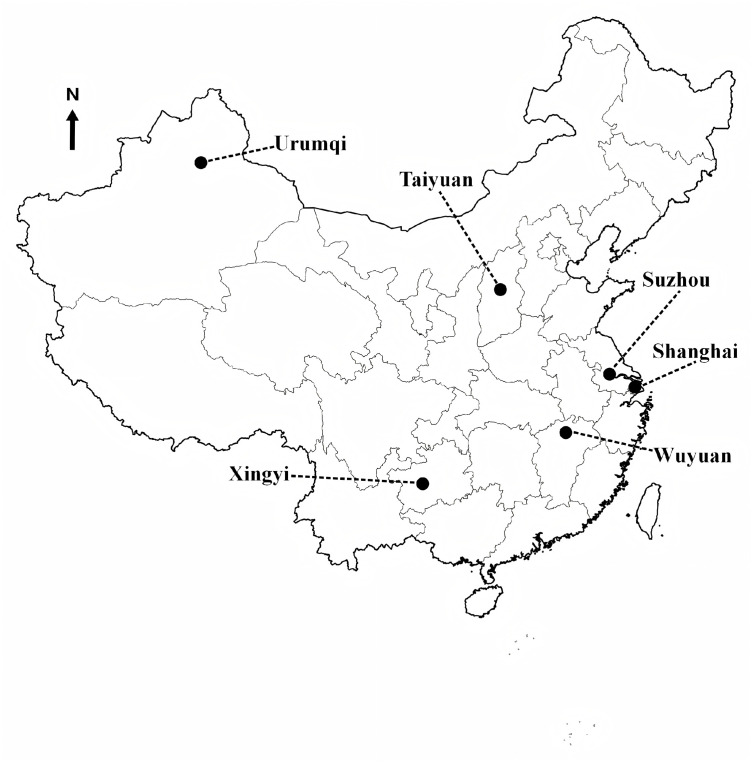
Distribution of sampling regions of adolescent participants in China. The base map was created using publicly available data from Natural Earth (https://www.naturalearthdata.com/), which is released into the public domain under the CC0 1.0 Universal Public Domain Dedication and is fully compatible with the CC BY 4.0 license.

Data for this nationally representative study of adolescent sleep quality were collected directly by the investigators and the research team through a cross-sectional questionnaire survey conducted between September and December 2023 in six cities in China: Shanghai, Suzhou, Taiyuan, Wuyuan, Xingyi, and Urumqi (Fig 1). The study protocol was approved by the Ethics Committee for Human Experimentation of East China Normal University (Approval No. HR761–2022). To ensure sample representativeness, participants were recruited using a stratified random cluster sampling method, ensuring approximately equal representation across gender and age groups First, accounting for socioeconomic factors, three secondary schools were randomly selected from each city-stratified by urban and rural locations-resulting in a total of 18 schools. Within each school, using the grade level as the primary sampling unit, intact classes were selected via a lottery method, yielding a total of 201 classes. Subsequently, students from these classes who were without reported physical or mental disabilities and provided informed consent were enrolled. Ultimately, the study included a total of 6,500 participants.

**Fig 2:**
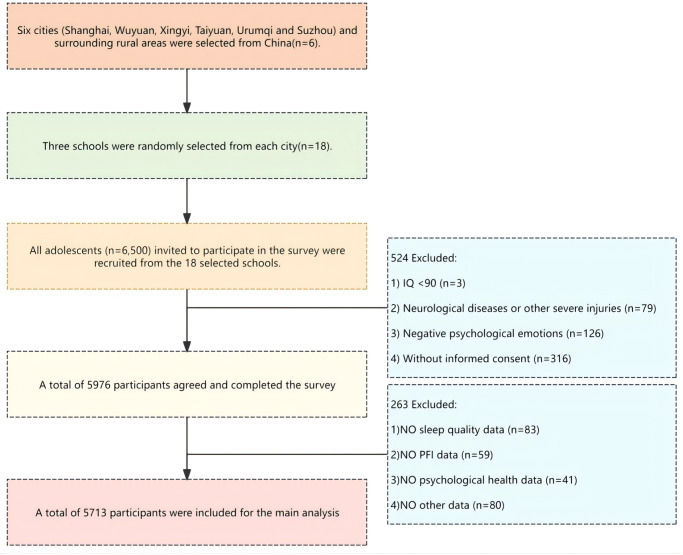
Sampling flow of adolescent participants in China.

After receiving approval from the participating schools, the research team informed participants and their parents/guardians of the study objectives and obtained written informed consent from all participants. All students’ names were digitally coded to protect confidentiality. The inclusion criteria were as follows: (1) Adolescents with an intelligence quotient (IQ) > 90 on the Wechsler Intelligence Scale (WIS) were included. In the WIS classification system, IQ scores above 90 fall within the “average” to “high-average” range. This threshold is consistent with prior studies in general populations, which commonly use IQ above 90 to ensure that participants can complete study tasks (e.g., the Pittsburgh Sleep Quality Index [PSQI] and mental health questionnaires) without introducing confounding due to cognitive limitations. Individuals with lower IQ scores may have difficulty completing or understanding the tasks, potentially compromising assessment accuracy. For participants whose scores were close to the cutoff (e.g., 85–89), study staff consulted teachers to further evaluate whether these students met the inclusion criteria. (2) Participants had no neurological disorders or other serious physical conditions (e.g., severe injury or disability). Because the study included physical fitness testing, all participants were screened for neurological disease or physical disability before study initiation to ensure safety. Students with such conditions were excluded, and the informed-consent form included a written item confirming the absence of the above conditions. (3) Participants showed no marked negative psychological or emotional states. After preliminary selection, researchers communicated with each participant’ s class teachers; because teachers are familiar with students’ daily behaviors and emotional status, they could provide valuable input. Before formal testing, teachers were consulted to assess whether students exhibited pronounced negative emotions (e.g., anxiety or depression). Questionnaires with a completion rate < 90% were considered to contain missing data and were excluded. Ultimately, 5,713 valid questionnaires were included, yielding an effective response rate of 87.9% (Fig 2).

### Measurements

#### Sleep quality.

Sleep quality was assessed using the Pittsburgh Sleep Quality Index (PSQI), which evaluates sleep quality during a typical school month [1]. The PSQI comprises 19 self-rated items and five bed-partner/roommate-rated items; the 19th self-rated item and the five other-rated items are not included in scoring. The remaining 18 items cover seven dimensions of sleep quality: subjective sleep quality, sleep latency, sleep duration, habitual sleep efficiency, sleep disturbances, need for sleep medications, and daytime dysfunction. Each component is scored from 0 to 3, with higher scores indicating more severe problems in that domain. Component scores are summed to generate a global PSQI score (range: 0–21), with higher total scores indicating poorer sleep quality. A global PSQI score ≤ 5 is considered indicative of good sleep quality, whereas a score > 5 indicates poor sleep quality [31]. In the present study, the PSQI demonstrated good internal consistency (Cronbach’ s α = 0.81), and Bartlett’ s test of sphericity was significant (KMO = 0.88, P < 0.01). Previous research has confirmed that the PSQI has satisfactory psychometric properties in adolescent samples [32,33]. In addition, the Chinese version of the PSQI has been validated and shown to be a reliable and valid instrument for assessing sleep quality [34,35].

#### Mental health.

Adolescents’ mental health status was assessed using the Brief Adolescent Mental Health Questionnaire. This instrument was derived from the psychological domain of the Multidimensional Sub-health Questionnaire of Adolescents (MSQA) developed by Qi Yuxiu et al [36]. The questionnaire covers three domains-emotional symptom, behavioural symptoms, and social adaptation difficulties-and contains 15 items. Each item is rated on a six-point scale according to symptom duration (1 = lasting > 3 months; 2 = lasting > 2 months; 3 = lasting > 1 month; 4 = lasting > 2 weeks; 5 = lasting > 1 week; 6 = none or lasting ≤ 1 week), with lower response categories indicating a longer duration of suboptimal mental health symptoms. Responses are scored from 1 to 6, yielding a total score ranging from 15 to 90; higher scores indicate better mental health. A total score at or above the 10th percentile (≥ P10) was classified as mentally healthy. This questionnaire was specifically designed to assess psychological symptoms in adolescents and has been used in multiple studies. In the present study, internal consistency was excellent (Cronbach’ s α = 0.96) [37–39].

#### Physical fitness.

Participants’ height and weight were measured in accordance with the requirements of the National Student Physical Fitness Standard (2014). During measurement, participants were barefoot and wore light clothing (a single top and trousers) while standing on the measuring device for assessment of height and weight. Height (m) and weight (kg) were recorded to one decimal place, and body mass index (BMI) was calculated as weight (kg) / [height (m)]².Adolescents completed tests of handgrip strength, standing broad jump, 50-m sprint, modified sit-and-reach, 20-s repeated straddle crossing, 30-s sit-ups, and the 20-m shuttle run test (20mSRT). Physical fitness testing was administered by trained teachers and student assistants. Participants were instructed to wear lightweight clothing, and the requirements and purpose of the tests were explained before assessment; testing proceeded only after informed consent was obtained.Scores from the seven fitness tests were standardized within gender- and age-specific groups to generate Z scores: Z = (observed value-mean) / SD. Z scores were summed to derive a Physical Fitness Index (PFI). For running-based measures, Z scores were reverse-coded because higher Z scores indicate poorer performance. For ease of interpretation, the raw PFI was further converted to a Z score; PFI Z ≥ 0 was classified as “good” and PFI Z scores < 0 as “poor.” [40].

#### MVPA and sedentary behaviors.

Physical activity status, sedentary time, and screen time were assessed using the Adolescent Physical Activity Questionnaire in the Guidelines for Evaluating the Physical Activity Levels of Children and Adolescents Between the Ages of 7 and 18 Years, published by the National Bureau of Disease Control and Prevention of China (WS/T 10008  −  2023) [41]. Physical activity information included the types of activities performed during physical education classes, as well as frequency, duration, and self-perceived intensity. Moderate-to-vigorous physical activity (MVPA) was defined based on metabolic equivalents (METs), with MVPA classified as ≥ 3 METs. Average daily MVPA time (min/day) was calculated as: weekly frequency * mean duration per session (min) / 7. The scale showed a test-retest reliability coefficient of 0.61.Sedentary time and screen time were collected by recording activity type, frequency, and duration. Screen time included four items: watching television/movies and using a computer, mobile phone, and tablet. Sedentary time included five items: completing paper-based homework, reading printed books, commuting to school by vehicle, sitting and chatting, and sitting in class. Average daily screen/sedentary time was calculated as: (frequency * duration for each behavior) / 7. The questionnaire demonstrated acceptable reliability and validity (test-retest reliability = 0.606; correlation coefficient = 0.689; all P < 0.01) [41].

### Statistical analyses

Statistical analyses were conducted using R (version 4.5.2) and SPSS Statistics (version 27; IBM Corp., Armonk, NY, USA). For descriptive statistics, normally distributed continuous variables are presented as mean ± standard deviation, non-normally distributed continuous variables as median (P25, P75), and categorical variables as percentages. Based on established cutoffs, the Pittsburgh Sleep Quality Index (PSQI) global score and component scores were dichotomized: a global PSQI score ≤ 5 indicated good sleep quality, whereas a score > 5 indicated poor sleep quality; for PSQI components, scores of 2–3 were classified as poor and scores of 0–1 as good. Continuous variables were compared between groups using the Wilcoxon rank-sum test (Mann-Whitney U test), and categorical variables were compared using the chi-square (χ²) test. Univariable logistic regression models were used to examine associations between dichotomized sleep-related outcomes (PSQI global score, subjective sleep quality, sleep latency, sleep duration, sleep disturbances, need of sleep medication, and daytime dysfunction) and potential correlates (gender, residence, PFI, BMI, mental health score, sedentary time, and screen time). For continuous predictors, variables were mean-centered and quadratic terms were evaluated to assess potential non-linear associations; quadratic terms were included only when statistically significant. For all predictors except age, gender, and residence, interactions between predictors and gender, as well as between predictors and residence, were tested. Subsequently, multivariable logistic regression analyses were performed to obtain P values and odds ratios (ORs) for all relevant factors and their interaction terms in relation to sleep-quality outcomes, while controlling for age. Two-sided P < 0.05 was considered statistically significant. Model diagnostics included the Brier score, discrimination metrics (ROC curve and AUC), and calibration assessment (calibration curve). Because the primary aim of this study was to identify and quantify risk and protective factors for adolescent sleep quality, no formal adjustment for multiple comparisons was applied; results that approached statistical significance should therefore be interpreted with caution.

## Results

### Participant demographic characteristics

A total of 5,713 participants were included in the demographic analysis, with an overall survey completion rate exceeding 85% (Table 1). Of these, 3,038 resided in urban areas and 2,675 in rural areas; 2,908 were male and 2,805 were female. The mean age of the sample was 15.11 ± 1.70 years. In the urban-rural comparison, urban adolescents had significantly higher BMI and sedentary time than rural adolescents (p < 0.05 and p < 0.001, respectively). Urban adolescents also showed higher PFI Z-scores, MVPA time, mental health scores, and screen time, although these differences did not reach statistical significance. In the gender-stratified comparison, all included demographic indicators showed highly significant differences between males and females. Male adolescents had significantly higher MVPA time, BMI, mental health scores, and screen time than female adolescents (all p < 0.001). In contrast, female adolescents had significantly higher sedentary time and PFI Z-scores than male adolescents (p = 0.001).

**Table 1:** Demographic characteristics in overall participants, by domicile and by gender.

Variable	Overall	Urban sample	Rural sample			Male sample	Female sample		
	M (P25, P75)	M (P25, P75)	M (P25, P75)	*Z*	*p*	M (P25, P75)	M (P25, P75)	*Z*	*p*
BMI	20.58 (18.56, 23.80)	20.80 (18.60, 24.02)	20.44 (18.51, 23.48)	2.50	**0.012**	20.96 (18.80, 24.61)	20.30 (18.34, 23.11)	6.91	**< 0.001**
PFI	0.20 (−1.54, 2.16)	0.37 (−1.23, 2.30)	−0.03 (−1.88, 1.93)	6.77	**< 0.001**	1.49 (−0.65, 3.36)	−0.62 (−2.02, 0.65)	27.22	**< 0.001**
MVPA (min)	62.86 (33.57, 105.71)	63.57 (34.29, 107.14)	61.43 (32.14, 103.93)	1.59	0.111	68.57 (35.71, 118.97)	55.71 (31.43, 92.86)	8.65	**< 0.001**
Sedentary time (h)	10.14 (7.83, 12.71)	10.34 (8.06, 12.91)	9.83 (7.48, 12.50)	5.28	**< 0.001**	9.93 (7.67, 12.66)	10.32 (8.00, 12.83)	−3.19	**0.001**
Screen time (h)	0.62 (0.21, 1.50)	0.64 (0.21, 1.50)	0.62 (0.18, 1.50)	0.97	0.333	0.71 (0.24, 1.67)	0.57 (0.19, 1.43)	4.92	**< 0.001**
Emotional symptoms	40.00 (33.00, 42.00)	40.00 (33.00, 42.00)	39.00 (34.00, 42.00)	—	—	40.00 (35.00, 42.00)	39.00 (32.00, 42.00)	—	—
Behavioral symptoms	24.00 (21.00, 24.00)	24.00 (21.00, 24.00)	24.00 (21.00, 24.00)	—	—	24.00 (22.00, 24.00)	24.00 (21.00, 24.00)	—	—
Social adaptation difficulties	23.00 (18.00, 24.00)	23.00 (18.00, 24.00)	23.00 (19.00, 24.00)	—	—	23.00 (19.00, 24.00)	23.00 (18.00, 24.00)	—	—
Psychological health	84.00 (73.00, 90.00)	85.00 (72.00, 90.00)	84.00 (74.00, 89.00)	0.24	0.812	85.00 (75.00, 90.00)	83.00 (70.00, 89.00)	8.09	**< 0.001**

Significant values are highlighted in bold; BMI, body mass index; PFI, physical fitness index; MVPA, moderate to vigorous physical activity.

### Sleep quality by region and by gender

Descriptive statistics of sleep quality showed that 33.71% of adolescents had poor sleep quality (Table 2). In urban-rural comparisons, urban adolescents had significantly better sleep quality than their rural counterparts, as indicated by lower global PSQI scores (p < 0.001). Across PSQI components, urban adolescents scored better than rural adolescents on four dimensions-subjective sleep quality, sleep latency, sleep duration, and sleep disturbances-with highly significant differences (all p < 0.001). Differences in daytime dysfunction and habitual sleep efficiency did not reach statistical significance, although urban adolescents still exhibited more favorable scores. The PSQI-based categorical classification showed a consistent pattern: the proportion of adolescents meeting the “good” sleep-quality criterion was significantly higher in urban than in rural areas (31.90% / 35.78%, χ^2^ = 9.41, p = 0.002).

**Table 2:** PSQI and component scores in all participants, by region and by gender.

Variable	Overall (n = 5713)	Urban sample (n = 3038)	Rural sample (n = 2675)	*Z*	*p*	Male sample (n = 2908)	Female sample (n = 2805)	*Z*	*p*
PSQI total score, M ± SD	4.56 ± 2.65	4.44 ± 2.70	4.70 ± 2.58	−4.52	**< 0.001**	4.20 ± 2.62	4.94 ± 2.63	−10.89	**< 0.001**
Subjective sleep quality, M ± SD	0.81 ± 0.73	0.78 ± 0.75	0.84 ± 0.71	−3.6	**< 0.001**	0.76 ± 0.74	0.86 ± 0.72	−5.58	**< 0.001**
Sleep latency, M ± SD	0.81 ± 0.91	0.77 ± 0.92	0.84 ± 0.90	−3.65	**< 0.001**	0.79 ± 0.90	0.83 ± 0.92	−1.53	0.126
Sleep duration, M ± SD	0.58 ± 0.69	0.53 ± 0.67	0.63 ± 0.70	−5.23	**< 0.001**	0.51 ± 0.66	0.64 ± 0.71	−6.91	**< 0.001**
Habitual sleep efficiency, M ± SD	0.00 ± 0.05	0.00 ± 0.05	0.00 ± 0.05	0.25	0.805	0.00 ± 0.05	0.00 ± 0.06	−0.57	0.567
Sleep disturbance, M ± SD	0.78 ± 0.61	0.75 ± 0.62	0.82 ± 0.60	−4.84	**< 0.001**	0.70 ± 0.61	0.87 ± 0.60	−10.37	**< 0.001**
Need for sleep medications, M ± SD	0.07 ± 0.37	0.08 ± 0.39	0.06 ± 0.36	2.22	**0.026**	0.05 ± 0.33	0.08 ± 0.42	−2.86	**0.004**
Daytime dysfunction, M ± SD	1.51 ± 1.00	1.52 ± 1.03	1.50 ± 0.96	0.61	0.542	1.37 ± 1.00	1.66 ± 0.97	−10.87	**< 0.001**
substandard, %				χ^2^	*p*			χ^2^	*p*
PSQI total	33.71	31.90	35.78	9.41	**0.002**	29.20	38.40	53.67	< 0.001
Subjective sleep quality	14.63	14.85	14.39	0.233	0.629	13.69	15.61	4.251	**0.039**
Sleep latency	21.21	19.72	22.92	8.709	**0.003**	20.56	21.89	1.501	0.221
Sleep duration	10.54	8.79	12.52	21.047	**< 0.001**	8.43	12.73	28.034	**< 0.001**
Habitual sleep efficiency	0	0	0	N/A	N/A	0	0	N/A	N/A
Sleep disturbance	8.86	8.26	9.53	2.845	0.092	6.91	10.87	27.757	**< 0.001**
Need for sleep medications	1.94	2.01	1.87	0.144	0.705	1.55	2.35	4.862	**0.027**
Daytime dysfunction	52.06	51.78	52.37	0.203	0.653	45.84	58.5	91.743	**< 0.001**

Significant values are highlighted in bold; PSQI, Pittsburgh Sleep Quality Index.

Substantial gender differences in sleep quality were also observed. Compared with males, female adolescents had poorer sleep quality. Females had significantly higher global PSQI scores than males (Z = −10.9, p < 0.001), and only 61.6% of females met the “good” sleep-quality criterion, which was significantly lower than the corresponding proportion in males (70.8%, χ^2^ = 53.67, p < 0.001). Across PSQI components, females scored higher (worse) than males on five dimensions-subjective sleep quality, sleep duration, sleep disturbances, need of sleep medication, and daytime dysfunction-and these differences were statistically significant (all p < 0.01). Differences in sleep latency and habitual sleep efficiency were not statistically significant, although females still had higher scores than males.

### Correlates of poor sleep quality

Univariable logistic regression models were constructed for the dichotomized PSQI global score and component outcomes (S1 Appendix). Among the PSQI components, habitual sleep efficiency showed an extremely skewed distribution: 99.72% of adolescents had sleep efficiency > 85%. Therefore, sleep efficiency was not used as a dependent variable in the logistic regression analyses. Univariable results indicated that urban adolescents had lower odds of poor outcomes for the PSQI global score, sleep latency, and sleep duration than rural adolescents. Female adolescents faced a higher burden of sleep problems: except for sleep latency, females had significantly higher odds of poor outcomes across all sleep indicators compared with males (P < 0.05, OR > 1). BMI the primary risk factor for sleep quality, with higher BMI associated with greater odds of poor outcomes for all sleep indicators (P < 0.05, OR > 1). In addition, longer sedentary time and greater screen time were also associated with poorer sleep quality. Mental health was the strongest protective factor; higher mental health scores significantly reduced the risk across all sleep outcomes (P < 0.05, OR < 1). Adolescents with higher PFI Z-scores had significantly lower odds of poor outcomes for the PSQI global score, sleep latency, and sleep disturbances. MVPA showed some favorable association with sleep quality, but the effect size was extremely small in this large sample. Quadratic-term analyses indicated non-linear associations with sleep outcomes only for MVPA, sedentary time, screen time, and mental health.

Multivariable logistic regression analyses showed that residence and gender remained significant determinants of adolescent sleep quality (Table 3). Urban adolescents had significantly lower odds of poor outcomes than rural adolescents for the PSQI global score, sleep latency, sleep duration, and sleep disturbances. For females, statistical significance was attenuated only for subjective sleep quality; for all other outcomes, females still had higher odds of poor sleep-related outcomes than males (P < 0.05, OR > 1).Sedentary time and screen time were significantly associated with poorer outcomes for the PSQI global score, subjective sleep quality, sleep latency, sleep duration, sleep disturbances, and daytime dysfunction (P < 0.05, OR > 1). Only for sleep duration did greater screen time appear to reduce the odds of poor sleep duration; however, a non-linear association was observed, such that once screen time exceeded a certain range, it again increased the risk of poor sleep duration.After adjusting for all demographic covariates, the associations of BMI, PFI, and MVPA with sleep quality were significantly attenuated; statistically significant effects remained only for the PSQI global score and use of sleep medication (P < 0.05). The significance of both linear and non-linear associations between MVPA and all outcomes disappeared, whereas the other quadratic terms were not materially affected by covariate adjustment.

**Table 3:** Sleep Quality: multivariable logistic regression models for the possibility of reporting a “Poor” score on the PSQI (scores >5) and six of the sleep factor components (scores >1).

Variable	PSQI Total		Subjective sleep quality		Sleep latency		Sleep duration		Sleep disturbance		sleep medications		Daytime dysfunction	
	OR (95% CI)	*p*	OR (95% CI)	*p*	OR (95% CI)	*p*	OR (95% CI)	*p*	OR (95% CI)	*p*	OR (95% CI)	*p*	OR (95% CI)	*p*
Age	1.25 (1.20, 1.30)	**< 0.001**	1.08 (1.03, 1.14)	**< 0.001**	0.98 (0.93, 1.02)	0.24	1.68 (1.58, 1.79)	**< 0.001**	1.00 (0.94, 1.07)	0.99	1.02 (0.90, 1.15)	0.78	1.21 (1.17, 1.26)	**< 0.001**
Residence	0.84 (0.74, 0.95)	**0.01**	0.95 (0.80, 1.14)	0.61	0.76 (0.66, 0.87)	**< 0.001**	0.77 (0.63, 0.94)	**0.01**	0.64 (0.50, 0.82)	**< 0.001**	1.03 (0.64, 1.67)	0.89	1.04 (0.93, 1.18)	0.48
Gender	1.42 (1.24, 1.62)	**< 0.001**	1.01 (0.84, 1.22)	0.92	0.97 (0.83, 1.12)	0.67	1.61 (1.30, 2.00)	**< 0.001**	1.46 (1.14, 1.88)	**< 0.001**	1.67 (1.02, 2.75)	**0.04**	1.64 (1.45, 1.86)	**< 0.001**
BMI	1.00 (0.97, 1.02)	0.77	1.01 (0.98, 1.04)	0.47	1.02 (1.00, 1.05)	0.08	0.99 (0.96, 1.03)	0.61	1.02 (0.99, 1.06)	0.17	1.07 (1.01, 1.14)	**0.02**	1.01 (0.99, 1.04)	0.31
PFI	0.88 (0.80, 0.98)	**0.02**	0.92 (0.81, 1.05)	0.21	0.94 (0.85, 1.05)	0.26	1.00 (0.84, 1.18)	0.99	0.89 (0.76, 1.04)	0.14	1.09 (0.74, 1.61)	0.68	0.93 (0.84, 1.02)	0.11
MVPA	1.00 (1.00, 1.00)	0.42	1.00 (1.00, 1.00)	0.28	1.00 (1.00, 1.00)	0.70	1.00 (1.00, 1.00)	0.24	1.00 (1.00, 1.00)	0.75	1.00 (0.99, 1.00)	0.35	1.00 (1.00, 1.00)	0.25
Sedentary time	1.02 (1.00, 1.05)	0.09	1.04 (1.00, 1.07)	**0.05**	1.01 (0.98, 1.04)	0.56	1.04 (1.00, 1.09)	**0.04**	1.01 (0.97, 1.05)	0.78	1.01 (0.92, 1.10)	0.87	1.05 (1.02, 1.07)	**< 0.001**
Screen time	1.15 (1.07, 1.25)	**< 0.001**	1.09 (0.99, 1.20)	0.09	1.07 (1.01, 1.13)	**0.02**	0.80 (0.71, 0.90)	**< 0.001**	1.11 (1.03, 1.19)	**0.01**	0.90 (0.72, 1.12)	0.35	1.12 (1.04, 1.21)	**< 0.001**
Mental health	0.93 (0.92, 0.94)	**< 0.001**	0.93 (0.92, 0.94)	**< 0.001**	0.95 (0.94, 0.95)	**< 0.001**	0.99 (0.98, 1.00)	**0.01**	0.93 (0.92, 0.94)	**< 0.001**	0.95 (0.94, 0.97)	**< 0.001**	0.94 (0.93, 0.94)	**< 0.001**
BMI by Residence	1.03 (1.00, 1.06)	**0.03**	1.01 (0.98, 1.04)	0.59	1.01 (0.98, 1.04)	0.51	1.03 (0.99, 1.07)	0.13	1.04 (1.00, 1.08)	**0.05**	0.99 (0.93, 1.05)	0.69	1.00 (0.98, 1.03)	0.76
PFI by Residence	1.08 (0.95, 1.23)	0.23	1.11 (0.95, 1.30)	0.21	1.03 (0.90, 1.18)	0.63	1.23 (1.01, 1.49)	**0.04**	0.89 (0.73, 1.09)	0.26	0.97 (0.64, 1.49)	0.91	1.13 (1.00, 1.27)	**0.05**
MVPA by Residence	1.00 (1.00, 1.00)	0.22	1.00 (1.00, 1.00)	0.06	1.00 (1.00, 1.00)	0.32	1.00 (1.00, 1.01)	**0.02**	1.00 (1.00, 1.00)	0.24	1.00 (0.99, 1.00)	0.27	1.00 (1.00, 1.00)	0.27
Sedentary time by Residence	1.00 (0.97, 1.03)	0.88	1.01 (0.97, 1.05)	0.69	0.99 (0.96, 1.03)	0.72	1.01 (0.97, 1.06)	0.57	1.01 (0.96, 1.06)	0.69	1.01 (0.92, 1.11)	0.87	0.99 (0.97, 1.02)	0.68
Screen time by Residence	0.95 (0.89, 1.03)	0.21	0.98 (0.90, 1.07)	0.71	0.96 (0.89, 1.03)	0.23	1.08 (0.98, 1.20)	0.13	0.94 (0.84, 1.04)	0.21	1.22 (0.96, 1.53)	0.10	1.01 (0.94, 1.09)	0.73
Mental health by Residence	0.98 (0.97, 0.99)	**< 0.001**	0.99 (0.98, 1.00)	0.15	0.99 (0.99, 1.00)	0.14	0.99 (0.98, 1.00)	0.08	0.99 (0.98, 1.00)	0.06	1.01 (0.99, 1.03)	0.29	0.99 (0.98, 1.00)	**< 0.001**
BMI by Gender	1.05 (1.02, 1.08)	**< 0.001**	1.08 (1.04, 1.11)	**< 0.001**	1.04 (1.01, 1.07)	**0.01**	1.07 (1.03, 1.11)	**< 0.001**	1.04 (1.00, 1.08)	**0.03**	1.02 (0.96, 1.09)	0.50	1.01 (0.98, 1.03)	0.62
PFI by Gender	1.18 (1.03, 1.36)	**0.02**	1.01 (0.85, 1.20)	0.92	0.99 (0.85, 1.15)	0.91	0.91 (0.74, 1.12)	0.39	1.23 (1.00, 1.52)	0.06	1.23 (0.80, 1.90)	0.35	1.26 (1.11, 1.44)	**< 0.001**
MVPA by Gender	1.00 (1.00, 1.00)	0.14	1.00 (1.00, 1.00)	0.61	1.00 (1.00, 1.00)	0.25	1.00 (0.99, 1.00)	**0.03**	1.00 (1.00, 1.00)	0.72	1.00 (1.00, 1.01)	0.53	1.00 (1.00, 1.00)	0.33
Sedentary time by Gender	0.98 (0.95, 1.01)	0.13	0.95 (0.92, 0.99)	**0.01**	0.97 (0.94, 1.01)	0.11	0.97 (0.93, 1.02)	0.20	0.97 (0.93, 1.02)	0.20	0.97 (0.88, 1.07)	0.52	0.99 (0.96, 1.02)	0.37
Screen time by Gender	0.94 (0.88, 1.01)	0.11	1.01 (0.93, 1.10)	0.85	0.99 (0.92, 1.07)	0.84	1.01 (0.91, 1.11)	0.90	0.95 (0.86, 1.05)	0.30	1.01 (0.82, 1.25)	0.92	0.96 (0.89, 1.03)	0.21
Mental health by Gender	1.00 (0.99, 1.01)	0.70	1.00 (0.99, 1.01)	0.44	1.00 (1.00, 1.01)	0.42	1.00 (0.99, 1.01)	0.88	1.00 (0.99, 1.01)	0.87	1.01 (1.00, 1.03)	0.16	1.00 (0.99, 1.01)	0.78
MVPA Quadratic	–	–	–	–	–	–	1.00 (1.00, 1.00)	0.24	–	–	–	–	1.00 (1.00, 1.00)	0.32
Sedentary time Quadratic	–	–	–	–	–	–	–	–	1.00 (1.00, 1.01)	**0.01**	–	–	1.00 (1.00, 1.00)	**0.01**
Screen time Quadratic	0.99 (0.98, 1.00)	**0.05**	1.00 (0.98, 1.01)	0.32	–	–	1.03 (1.02, 1.04)	**< 0.001**	–	–	–	–	0.99 (0.98, 1.00)	**0.04**
Mental health Quadratic	1.00 (1.00, 1.00)	**< 0.001**	1.00 (1.00, 1.00)	**< 0.001**	1.00 (1.00, 1.00)	**< 0.001**	–	–	1.00 (1.00, 1.00)	**< 0.001**	–	–	1.00 (1.00, 1.00)	**< 0.001**

Significant values are highlighted in bold; BMI, body mass index; PFI, Physical fitness index; MVPA, Moderate to vigorous physical activity; CI, confidence interval; OR, odds ratio; PSQI, Pittsburgh Sleep Quality Index.

**Fig 3:**
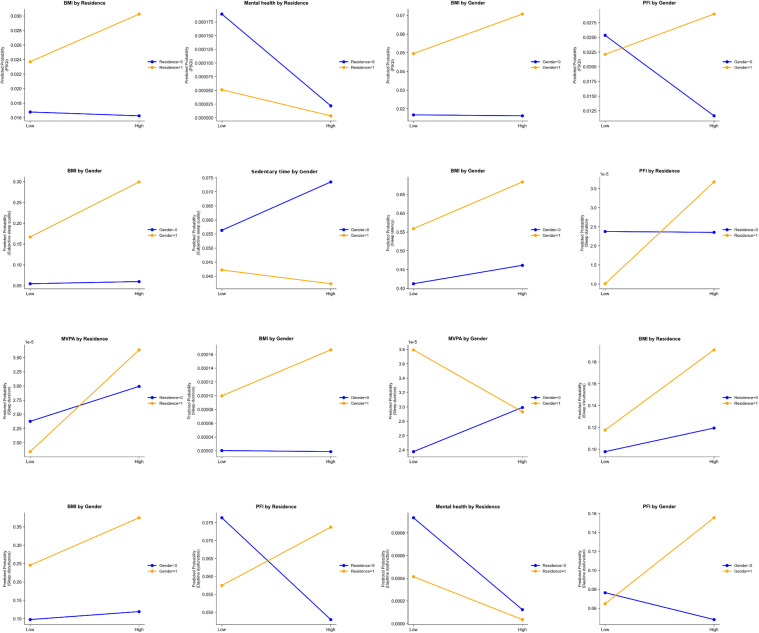
Simple slope plots of the 16 significant interaction terms from the multivariable logistic regression model.

Regarding interaction terms, the regression results for residence-based interactions indicated that residence moderated the associations of BMI, PFI, MVPA, and mental health with sleep quality (Fig 3). The adverse effects of higher BMI on the PSQI global score and sleep disturbances were more pronounced among urban adolescents. The protective effects of better mental health on the PSQI global score and daytime dysfunction were stronger among rural adolescents. In urban adolescents, increases in PFI and MVPA were more likely to be associated with insufficient sleep duration and daytime dysfunction. Gender also moderated the associations of BMI, PFI, MVPA, and sedentary time with sleep quality. Sleep quality in female adolescents was more sensitive to variations in BMI; specifically, the negative associations of higher BMI with the PSQI global score, subjective sleep quality, sleep latency, sleep duration, and sleep disturbances were significantly stronger in females than in males. Moreover, the protective association of higher PFI with the PSQI global score was observed only in males; in contrast, higher PFI was associated with a greater likelihood of poor sleep quality and daytime dysfunction among females (P < 0.05). Finally, the detrimental association of sedentary time with sleep quality was more pronounced in males (P < 0.05).

## Discussion

This cross-sectional study of Chinese adolescents aged 13–18 years examined differences in sleep quality across residences and between gender, as well as factors associated with poorer sleep quality. The results supported only hypotheses 2 and 3: sleep quality was significantly poorer among rural adolescents than among urban adolescents, and significantly poorer among females than among males. Logistic regression analyses of sleep outcomes and demographic/behavioral indicators indicated that mental health, physical fitness, and sedentary behaviors were all associated with adolescent sleep quality to varying degrees, and that the strength of these associations was moderated by residence and gender.

Overall, the findings regarding sleep quality among Chinese adolescents were relatively encouraging: in this study, only 34% of participants reported suboptimal sleep quality over the past month. Among PSQI components, sleep duration was generally low. Although only 10.54% of adolescents had poor sleep duration based on the PSQI criterion (component score ≥ 2), more than 46.70% reported sleeping < 7 h per night, below the duration recommended by the U.S. National Sleep Foundation (NSF) [42]. This pattern is broadly consistent with prior studies of Chinese adolescents, in which the prevalence of insufficient sleep has ranged from 30% to 85% and has generally exceeded estimates reported in other countries (14%−50.0%) [18,23,43–50]. Differences in sleep duration may be partly attributable to China’s distinctive cultural and educational context. Chinese education, influenced by Confucian traditions, places strong emphasis on academic achievement. accordingly, parents and teachers often prioritize academic performance, and many adolescents tend to sacrifice sleep for studying [51,52].

In addition, 52.06% of adolescents had poor daytime dysfunction (component score ≥ 2). Higher daytime dysfunction scores indicate that adolescents may experience symptoms such as excessive daytime sleepiness, fatigue, inattention, and low mood [32]. Many studies have similarly reported persistently high rates of daytime dysfunction in adolescent populations [49,53,54]. The primary driver of this pattern is insufficient total sleep time, which is consistent with the short sleep duration observed in the present sample [53,55,56]. Moreover, studies have identified several additional contributors to daytime dysfunction during adolescence, including rapidly increasing use of digital technologies and media, greater consumption of caffeine and energy drinks, and pubertal hormonal changes and circadian rhythm disruption [57–61].

Previous research has posited that urban residents are typically exposed to a higher density of sleep-related risk factors such as noise and light pollution. In addition, compared with rural areas, a faster pace of life and greater psychosocial stress in cities may adversely affect sleep quality [17,19]. Wittmann et al., drawing on the social jetlag hypothesis, also reported that rural areas with lower levels of industrialization tend to exhibit smaller misalignment between social time and natural time, which may better support biologically driven sleep needs [62–65]. However, the findings of the present study contradict prior research, rural adolescents had higher odds of poor outcomes in sleep quality, sleep latency, sleep duration, sleep disturbances, and daytime dysfunction. Several factors may explain this pattern. First, as educational norms and broader sociocultural values diffuse, the urban-rural gap in academic pressure among adolescents may be narrowing. Second, contemporary China has experienced large-scale rural-to-urban migration. Rural adolescents may more often face poorer household socioeconomic conditions and stress related to family separation, which can lead them to assume social responsibilities and household duties earlier than their urban peers. Rural adolescents may also contend with earlier school schedules and less favorable commuting conditions, both of which could further erode sleep time. Finally, living in rural areas may entail more limited access to healthcare services and sleep-hygiene education, which may also contribute to poorer sleep quality [17,19].

With respect to gender differences in adolescent sleep quality, our findings are consistent with previous research, showing that sleep quality is significantly poorer in females than in males [21,66–68]. Several mechanisms may contribute to this pattern. First, gender-specific hormonal changes during puberty may significantly influence sleep. Rising testosterone levels in males may promote a greater proportion of deep sleep, whereas female adolescents entering reproductive maturation experience cyclical fluctuations in estrogen and progesterone, which may increase sleep fragmentation, emotional variability, and circadian dysregulation, thereby directly or indirectly impairing sleep quality [61,69,70].Second, sociocultural pressures may exert a stronger influence on sleep in female adolescents. Females tend to spend more time using social media at night and may experience greater pressure toward academic perfectionism, which can contribute to delayed bedtimes and reduced sleep efficiency [71–74]. Female adolescents may also exhibit poorer sleep-hygiene practices while being more likely to consume caffeinated beverages, factors that could further shorten sleep duration relative to males [23]. Finally, mental health problems such as anxiety and depressive symptoms are more prevalent among females, which may further amplify gender disparities in sleep quality [75].

In this study, we selected six indicators spanning physical fitness, mental health, and sedentary behaviors – BMI, MVPA, PFI, mental health score, sedentary time, and screen time-and examined their associations with sleep quality among Chinese adolescents. With respect to physical health, better fitness was associated with better sleep quality, consistent with prior evidence showing that higher fitness levels are linked to lower PSQI scores (i.e., better sleep) [76–79]. From a physiological perspective, cardiorespiratory fitness, a core component of physical fitness, may enhance vagal activity and reduce sympathetic tone, thereby decreasing nocturnal awakenings and improving sleep stability [80,81]. In addition, regular exercise may stabilize circadian rhythms, regulate melatonin secretion, and strengthen the sleep-wake regulatory system, thereby improving sleep onset and sleep efficiency [82,83].In contrast, our results suggested that MVPA was negatively associated with sleep duration in Chinese adolescents. Although this linear association was no longer significant after adjustment for other covariates, this finding appears counterintuitive. Brand et al. reported that physical activity benefits adolescents’ total sleep time, sleep latency, and sleep efficiency [84]. However, some studies have found no significant association between increased MVPA alone and improvements in sleep duration or sleep efficiency [85]. The relationship between physical activity and sleep may depend on exercise modality, timing, and type. For example, high-intensity exercise or poorly timed activity may cause excessive sympathetic activation, which could transiently delay sleep onset [86].

Regarding sedentary behaviors, longer screen time was associated with significantly higher odds of poor outcomes in the PSQI global score, sleep latency, sleep disturbances, and daytime dysfunction. Moreover, based on the quadratic-term results, these adverse associations may intensify once screen time exceeds a certain threshold. The negative impact of screen time on sleep has been widely documented, including shorter sleep duration, delayed bedtimes, poorer sleep quality, and greater daytime dysfunction [26,27,57–59]. Proposed mechanisms can be summarized as follows. First, light exposure is a primary zeitgeber for circadian regulation, and any additional nocturnal light-particularly in the blue spectrum-may increase alertness, reduce pre-sleep sleepiness, disrupt sleep architecture, delay circadian timing, and even increase risks of depression and all-cause mortality [87–89]. Second, using electronic screens before bedtime can displace sleep by extending leisure time [90]. Third, adolescents with sleep difficulties may be more likely to use electronic devices at night in an attempt to facilitate sleep [86].

Mental health emerged as the most important protective factor for adolescent sleep quality, with stronger protective associations observed at higher mental health scores. This finding aligns with a substantial body of prior research showing that mental health is closely linked to multiple components of sleep quality [68,91–94]. In the present study, the mental health score comprised emotional symptom, behavioural symptoms, and social adaptation difficulties-three domains that have each been shown to be strongly associated with sleep quality [95–97].

By incorporating interaction terms in the logistic regression models, this study revealed that residence and gender moderated the relationships between sleep quality and five key indicators: mental health score, PFI, MVPA, BMI, and sedentary time. Overall, the adverse effects of these risk factors were more pronounced among urban adolescents and among females. This pattern is consistent with the “urban penalty” framework proposed in recent literature [98,99]. Sleep quality in urban adolescents may already be approaching a tolerance threshold because of higher academic pressure, greater caffeine intake, and more intense noise and light pollution, rendering them more vulnerable to additional risk exposures [100–104]. Moreover, early-life urban residence has been positively associated with anxiety and affective disorders as well as with obesity and chronic metabolic diseases [105,106]. In the present study, urban adolescents had significantly higher BMI than rural adolescents, and this higher baseline level may partly explain why their sleep quality was more susceptible to risk factors. The gender differences also align with this framework. Compared with males, females tend to have poorer sleep-hygiene practices and are more vulnerable to psychological factors such as anxiety and depression, which may bring female adolescents closer to their tolerance threshold for sleep-disrupting exposures [23,107]. In addition, hormonal changes during reproductive maturation may increase susceptibility to obesity and to obesity-related secondary risks (e.g., body-image anxiety) [108,109]. In our sample, females also had significantly lower fitness levels and less time spent in physical activity than males; these lower baseline levels may limit the extent to which protective factors can counteract declining sleep quality.

In addition, it should be noted that in the adolescent population, sleep quality problems often do not occur in isolation, but rather exist in interrelated and clustered forms. Findings from Mezzofranco et al. on sleep disturbances in school-aged children indicated that the factors affecting sleep quality and sleep continuity are multidimensional in nature [110]. Buysse et al. similarly proposed that sleep health should be understood as a multidimensional pattern, rather than as the result of a single symptom or indicator [111]. Against this background, the influences of residence, gender, and mental health status—each of which is an important factor associated with sleep quality—on sleep duration, sleep disturbances, subjective sleep quality, and daytime functioning may not be entirely independent, but may instead exert integrated effects on clusters of related indicators. This integrated perspective may help explain why multiple dimensions of the Pittsburgh Sleep Quality Index (PSQI) showed unfavorable patterns within the same population, and why an increase in a given protective factor may lead to an overall improvement in sleep quality.

### Limitation of this study

This study has several strengths. By simultaneously examining the relationships between sleep quality and multiple physical- and mental-health-related factors in a large multi-city sample, and further investigating the moderating roles of residence and gender in these associations, the present study was designed to provide a more comprehensive perspective on adolescent sleep health in China. The findings may contribute to a better understanding of sleep health among Chinese adolescents and provide a reference for developing sleep-health promotion strategies that are more sensitive to regional and gender-related differences. Several limitations should be noted.

First, the cross-sectional design does not support causal inference. Although this study identified significant associations between sleep quality and multiple demographic, behavioral, and mental-health factors, the cross-sectional nature of the data limits any inference regarding the directionality of these relationships. Accordingly, it remains unclear whether these factors influenced sleep quality, whether poor sleep quality affected these factors in turn, or whether bidirectional relationships may exist.

Second, most variables, including sleep quality and related behavioral indicators, were assessed using self-reported questionnaires, which may have introduced recall bias, reporting bias, and social desirability bias. In addition, the lack of objective sleep-related measures, such as quantitative indicators derived from accelerometry or wearable devices (e.g., sleep duration and sleep midpoint), may have reduced the precision of sleep assessment to some extent. Therefore, the associations observed in the present study should be interpreted with caution. Future research should incorporate objective sleep measurements and adopt longitudinal designs to further clarify these relationships.

Third, screen time and sedentary time were assessed as total daily duration. Analyzing these variables on the basis of cumulative time across the entire day may have diluted their observed associations with sleep quality, particularly when the timing of exposure is likely to be important. In addition, this study did not specifically measure several sleep-hygiene and bedroom-environment factors that may influence sleep quality, including pre-sleep caffeine intake, bedtime reading habits, the presence and use of electronic devices in the bedroom before sleep (e.g., computers, televisions, and mobile phones), and whether siblings or roommates were present during sleep. These unmeasured factors may have influenced some of the observed associations. Future studies should collect these bedtime-specific exposures in a more targeted manner to clarify their relationships with adolescent sleep quality.

## Conclusions

In this cross-sectional study of Chinese adolescents aged 13–18 years, Chinese adolescents living in rural areas and female adolescents experience a greater burden of poor sleep quality. Mental health and physical fitness appear to be protective factors for sleep quality, whereas BMI, sedentary time, and screen time are risk factors among Chinese adolescents. Moreover, these associations are moderated by residence and gender, such that sleep quality among females and urban adolescents is more susceptible to the adverse effects of these risk factors.

## Supporting information

S1 Appendix Sleep Quality: single predictor logistic regression models for the possibility of reporting a “Poor” score on the PSQI (scores >5) and six of the sleep factor components (scores >1). (DOCX)

S2 Appendix Raw data. (XLSX)

## References

[1] Buysse DJ, Reynolds CF 3rd, Monk TH, Berman SR, Kupfer DJ. The Pittsburgh Sleep Quality Index: a new instrument for psychiatric practice and research. Psychiatry Res. 1989;28(2):193–213. doi: 10.1016/0165-1781(89)90047-4 2748771

[2] Cheng G, Zhou H, Zhu Q. Why are adolescents sleep deprived? Research based on data from the China Education Panel Survey. Educational Science Research. 2022;(03):42–50.

[3] Hirshkowitz M, Whiton K, Albert SM, Alessi C, Bruni O, DonCarlos L, et al. National Sleep Foundation’s sleep time duration recommendations: methodology and results summary. Sleep Health. 2015;1(1):40–3. doi: 10.1016/j.sleh.2014.12.010 29073412

[4] Tononi G, Cirelli C. Sleep function and synaptic homeostasis. Sleep Med Rev. 2006;10(1):49–62. doi: 10.1016/j.smrv.2005.05.002 16376591

[5] Hayashino Y, Yamazaki S, Takegami M, Nakayama T, Sokejima S, Fukuhara S. Association between number of comorbid conditions, depression, and sleep quality using the Pittsburgh Sleep Quality Index: results from a population-based survey. Sleep Med. 2010;11(4):366–71. doi: 10.1016/j.sleep.2009.05.021 20219425

[6] Hoevenaar-Blom MP, Spijkerman AMW, Kromhout D, van den Berg JF, Verschuren WMM. Sleep duration and sleep quality in relation to 12-year cardiovascular disease incidence: the MORGEN study. Sleep. 2011;34(11):1487–92. doi: 10.5665/sleep.1382 22043119 PMC3198203

[7] Coughlin JW, Smith MT. Sleep, obesity, and weight loss in adults: is there a rationale for providing sleep interventions in the treatment of obesity? Int Rev Psychiatry. 2014;26(2):177–88. doi: 10.3109/09540261.2014.911150 24892893

[8] Kuo SI-C, Updegraff KA, Zeiders KH, McHale SM, Umaña-Taylor AJ, De Jesús SAR. Mexican American adolescents’ sleep patterns: contextual correlates and implications for health and adjustment in young adulthood. J Youth Adolesc. 2015;44(2):346–61. doi: 10.1007/s10964-014-0156-1 25047598 PMC4294970

[9] Lallukka T, Podlipskytė A, Sivertsen B, Andruškienė J, Varoneckas G, Lahelma E, et al. Insomnia symptoms and mortality: a register-linked study among women and men from Finland, Norway and Lithuania. J Sleep Res. 2016;25(1):96–103. doi: 10.1111/jsr.12343 26420582

[10] Cappuccio FP, D’Elia L, Strazzullo P, Miller MA. Sleep duration and all-cause mortality: a systematic review and meta-analysis of prospective studies. Sleep. 2010;33(5):585–92. doi: 10.1093/sleep/33.5.585 20469800 PMC2864873

[11] Grandner MA, Smith TE, Jackson N, Jackson T, Burgard S, Branas C. Geographic distribution of insufficient sleep across the United States: a county-level hotspot analysis. Sleep Health. 2015;1(3):158–65. doi: 10.1016/j.sleh.2015.06.003 26989761 PMC4790125

[12] Grandner MA, Patel NP, Gehrman PR, Perlis ML, Pack AI. Problems associated with short sleep: bridging the gap between laboratory and epidemiological studies. Sleep Med Rev. 2010;14(4):239–47. doi: 10.1016/j.smrv.2009.08.001 19896872 PMC2888649

[13] Grandner MA, Patel NP, Gehrman PR, Xie D, Sha D, Weaver T, et al. Who gets the best sleep? Ethnic and socioeconomic factors related to sleep complaints. Sleep Med. 2010;11(5):470–8. doi: 10.1016/j.sleep.2009.10.006 20388566 PMC2861987

[14] Samson DR, Crittenden AN, Mabulla IA, Mabulla AZP. The evolution of human sleep: Technological and cultural innovation associated with sleep-wake regulation among Hadza hunter-gatherers. J Hum Evol. 2017;113:91–102. doi: 10.1016/j.jhevol.2017.08.005 29054171

[15] Hale L, Emanuele E, James S. Recent Updates in the Social and Environmental Determinants of Sleep Health. Curr Sleep Med Rep. 2015;1(4):212–7. doi: 10.1007/s40675-015-0023-y 27540510 PMC4987093

[16] Jenner M. At day’s close: A history of nighttime. Oxford University Press. 2007.

[17] Xiang Y-T, Ma X, Cai Z-J, Li S-R, Xiang Y-Q, Guo H-L, et al. The prevalence of insomnia, its sociodemographic and clinical correlates, and treatment in rural and urban regions of Beijing, China: a general population-based survey. Sleep. 2008;31(12):1655–62. doi: 10.1093/sleep/31.12.1655 19090321 PMC2603488

[18] Meng LP, Liu AL, Hu X, Zhang Q, Du SM, Fang HY. Report on childhood obesity in China (10): association of sleep duration with obesity. Biomed Environ Sci. 2012;25(2):133–40. doi: 10.3967/0895-3988.2012.02.002 22998818

[19] Tang J, Liao Y, Kelly BC, Xie L, Xiang Y-T, Qi C, et al. Gender and Regional Differences in Sleep Quality and Insomnia: A General Population-based Study in Hunan Province of China. Sci Rep. 2017;7:43690. doi: 10.1038/srep43690 28262807 PMC5337959

[20] Mong JA, Cusmano DM. Sex differences in sleep: impact of biological sex and sex steroids. Philos Trans R Soc Lond B Biol Sci. 2016;371(1688):20150110. doi: 10.1098/rstb.2015.0110 26833831 PMC4785896

[21] Hysing M, Pallesen S, Stormark KM, Lundervold AJ, Sivertsen B. Sleep patterns and insomnia among adolescents: a population-based study. J Sleep Res. 2013;22(5):549–56. doi: 10.1111/jsr.12055 23611716

[22] Zhang J, Chan NY, Lam SP, Li SX, Liu Y, Chan JWY, et al. Emergence of Sex Differences in Insomnia Symptoms in Adolescents: A Large-Scale School-Based Study. Sleep. 2016;39(8):1563–70. doi: 10.5665/sleep.6022 27091537 PMC4945316

[23] Galland BC, Gray AR, Penno J, Smith C, Lobb C, Taylor RW. Gender differences in sleep hygiene practices and sleep quality in New Zealand adolescents aged 15 to 17 years. Sleep Health. 2017;3(2):77–83. doi: 10.1016/j.sleh.2017.02.001 28346161

[24] Johnson EO, Roth T, Schultz L, Breslau N. Epidemiology of DSM-IV insomnia in adolescence: lifetime prevalence, chronicity, and an emergent gender difference. Pediatrics. 2006;117(2):e247–56. doi: 10.1542/peds.2004-2629 16452333

[25] Wade TJ, Cairney J, Pevalin DJ. Emergence of gender differences in depression during adolescence: national panel results from three countries. J Am Acad Child Adolesc Psychiatry. 2002;41(2):190–8. doi: 10.1097/00004583-200202000-00013 11837409

[26] Cain N, Gradisar M. Electronic media use and sleep in school-aged children and adolescents: A review. Sleep Med. 2010;11(8):735–42. doi: 10.1016/j.sleep.2010.02.006 20673649

[27] Bartel KA, Gradisar M, Williamson P. Protective and risk factors for adolescent sleep: a meta-analytic review. Sleep Med Rev. 2015;21:72–85. doi: 10.1016/j.smrv.2014.08.002 25444442

[28] Rosenberg R, Czeisler C, Gradisar M, Hale L, Harvey A, Wolfson A. Annual Sleep in America Poll Exploring Connections with Communications Technology Use and Sleep. National Sleep Foundation. 2011. 10.5664/jcsm.3272 PMC3836340 24340291

[29] Calamaro CJ, Mason TBA, Ratcliffe SJ. Adolescents living the 24/7 lifestyle: effects of caffeine and technology on sleep duration and daytime functioning. Pediatrics. 2009;123(6):e1005-10. doi: 10.1542/peds.2008-3641 19482732

[30] Bryant Ludden A, Wolfson AR. Understanding adolescent caffeine use: connecting use patterns with expectancies, reasons, and sleep. Health Educ Behav. 2010;37(3):330–42. doi: 10.1177/1090198109341783 19858312

[31] Chen G, Xiang H, Mao Z, Huo W, Guo Y, Wang C, et al. Is long-term exposure to air pollution associated with poor sleep quality in rural China? Environ Int. 2019;133(Pt B):105205. doi: 10.1016/j.envint.2019.105205 31639600 PMC6853164

[32] de la Vega R, Tomé-Pires C, Solé E, Racine M, Castarlenas E, Jensen MP, et al. The Pittsburgh Sleep Quality Index: Validity and factor structure in young people. Psychol Assess. 2015;27(4):e22-7. doi: 10.1037/pas0000128 26653055

[33] Raniti MB, Waloszek JM, Schwartz O, Allen NB, Trinder J. Factor structure and psychometric properties of the Pittsburgh Sleep Quality Index in community-based adolescents. Sleep. 2018;41(6). doi: 10.1093/sleep/zsy066 29608755

[34] Tang J, Liao Y, He H, Deng Q, Zhang G, Qi C, et al. Sleeping problems in Chinese illicit drug dependent subjects. BMC Psychiatry. 2015;15:28. doi: 10.1186/s12888-015-0409-x 25884573 PMC4337091

[35] Tsai P-S, Wang S-Y, Wang M-Y, Su C-T, Yang T-T, Huang C-J, et al. Psychometric evaluation of the Chinese version of the Pittsburgh Sleep Quality Index (CPSQI) in primary insomnia and control subjects. Qual Life Res. 2005;14(8):1943–52. doi: 10.1007/s11136-005-4346-x 16155782

[36] Tao FB, Hu CL, Sun YH, Hao JH. The development and application of multidimensional sub-health questionnaire of adolescents (MSQA). Chinese Journal of Disease Control & Prevention. 2008;(04):309–14.

[37] Cao H, Qian Q, Weng T, Yuan C, Sun Y, Wang H, et al. Screen time, physical activity and mental health among urban adolescents in China. Prev Med. 2011;53(4–5):316–20. doi: 10.1016/j.ypmed.2011.09.002 21933680

[38] Wan Y-H, Xu S-J, Chen J, Hu C-L, Tao F-B. Longitudinal effects of psychological symptoms on non-suicidal self-injury: a difference between adolescents and young adults in China. Soc Psychiatry Psychiatr Epidemiol. 2015;50(2):237–47. doi: 10.1007/s00127-014-0917-x 24974078

[39] Wu X, Tao S, Zhang Y, Zhang S, Tao F. Low physical activity and high screen time can increase the risks of mental health problems and poor sleep quality among Chinese college students. PLoS One. 2015;10(3):e0119607. doi: 10.1371/journal.pone.0119607 25786030 PMC4364939

[40] Hartz J, Yingling L, Ayers C, Adu-Brimpong J, Rivers J, Ahuja C, et al. Clustering of Health Behaviors and Cardiorespiratory Fitness Among U.S. Adolescents. J Adolesc Health. 2018;62(5):583–90. doi: 10.1016/j.jadohealth.2017.11.298 29477492 PMC5930079

[41] Shang W, Yin X, Wang J, Hong J, Shi L, Guo J. Relationship between sedentary behavior, cardiorespiratory fitness, and executive function in adolescents. Chinese Journal of School Health. 2024;45(03):330–4. doi: 10.16835/j.cnki.1000-9817.2024091

[42] Hirshkowitz M, Whiton K, Albert SM, Alessi C, Bruni O, DonCarlos L, et al. National Sleep Foundation’s updated sleep duration recommendations: final report. Sleep Health. 2015;1(4):233–43. doi: 10.1016/j.sleh.2015.10.004 29073398

[43] An Y, Ji X, Zhou L, Liu J. Sleep and subjective well-being among Chinese adolescents: resilience as a mediator. Asian Journal of Social Health and Behavior. 2023;6(3).

[44] Zhang B, Hao Y, Zhou J, Jia F, Li X, Tang Y, et al. The association between sleep patterns and overweight/obesity in Chinese children: a cross-sectional study. Neuropsychiatr Dis Treat. 2015;11:2209–16. doi: 10.2147/NDT.S90838 26346134 PMC4556246

[45] Wang H, Hu R, Du H, Fiona B, Zhong J, Yu M. The relationship between sleep duration and obesity risk among school students: a cross-sectional study in Zhejiang, China. Nutr Metab (Lond). 2018;15:48. doi: 10.1186/s12986-018-0285-8 30002720 PMC6038205

[46] Michels N, Verbeiren A, Ahrens W, De Henauw S, Sioen I. Children’s sleep quality: relation with sleep duration and adiposity. Public Health. 2014;128(5):488–90. doi: 10.1016/j.puhe.2014.02.003 24694898

[47] Miguez MJ, Bueno D, Perez C. Disparities in sleep health among adolescents: the role of sex, age, and migration. Sleep Disord. 2020;2020:5316364. doi: 10.1155/2020/5316364 32089893 PMC7024093

[48] Khan MKA, Chu YL, Kirk SFL, Veugelers PJ. Are sleep duration and sleep quality associated with diet quality, physical activity, and body weight status? A population-based study of Canadian children. Can J Public Health. 2015;106(5):e277–82. doi: 10.17269/cjph.106.4892 26451988 PMC6972129

[49] Otsuka Y, Itani O, Nakajima S, Kaneko Y, Suzuki M, Kaneita Y. Impact of chronotype, insomnia symptoms, sleep duration, and electronic devices on nonrestorative sleep and daytime sleepiness among Japanese adolescents. Sleep Med. 2023;110:36–43. doi: 10.1016/j.sleep.2023.07.030 37531897

[50] Park JH, Yoo J-H, Kim SH. Associations between non-restorative sleep, short sleep duration and suicidality: findings from a representative sample of Korean adolescents. Psychiatry Clin Neurosci. 2013;67(1):28–34. doi: 10.1111/j.1440-1819.2012.02394.x 23279748

[51] Zhao X, Selman RL, Haste H. Academic stress in Chinese schools and a proposed preventive intervention program. Cogent Education. 2015;2(1):1000477. doi: 10.1080/2331186x.2014.1000477

[52] Wang L, Zhang Y. An extended version of the theory of planned behaviour: the role of self-efficacy and past behaviour in predicting the physical activity of Chinese adolescents. J Sports Sci. 2016;34(7):587–97. doi: 10.1080/02640414.2015.1064149 26148128

[53] Gradisar M, Gardner G, Dohnt H. Recent worldwide sleep patterns and problems during adolescence: a review and meta-analysis of age, region, and sleep. Sleep Med. 2011;12(2):110–8. doi: 10.1016/j.sleep.2010.11.008 21257344

[54] Kaneita Y, Ohida T, Osaki Y, Tanihata T, Minowa M, Suzuki K, et al. Insomnia among Japanese adolescents: a nationwide representative survey. Sleep. 2006;29(12):1543–50. doi: 10.1093/sleep/29.12.1543 17252885

[55] Owens J, Adolescent Sleep Working Group, Committee on Adolescence. Insufficient sleep in adolescents and young adults: an update on causes and consequences. Pediatrics. 2014;134(3):e921–32. doi: 10.1542/peds.2014-1696 25157012 PMC8194472

[56] Fallone G, Owens JA, Deane J. Sleepiness in children and adolescents: clinical implications. Sleep Med Rev. 2002;6(4):287–306. doi: 10.1053/smrv.2001.0192 12531133

[57] Carter B, Rees P, Hale L, Bhattacharjee D, Paradkar MS. Association Between Portable Screen-Based Media Device Access or Use and Sleep Outcomes: A Systematic Review and Meta-analysis. JAMA Pediatr. 2016;170(12):1202–8. doi: 10.1001/jamapediatrics.2016.2341 27802500 PMC5380441

[58] Munezawa T, Kaneita Y, Osaki Y, Kanda H, Minowa M, Suzuki K, et al. The association between use of mobile phones after lights out and sleep disturbances among Japanese adolescents: a nationwide cross-sectional survey. Sleep. 2011;34(8):1013–20. doi: 10.5665/SLEEP.1152 21804663 PMC3138156

[59] Hale L, Guan S. Screen time and sleep among school-aged children and adolescents: a systematic literature review. Sleep Med Rev. 2015;21:50–8. doi: 10.1016/j.smrv.2014.07.007 25193149 PMC4437561

[60] Crowley SJ, Acebo C, Carskadon MA. Sleep, circadian rhythms, and delayed phase in adolescence. Sleep Med. 2007;8(6):602–12. doi: 10.1016/j.sleep.2006.12.002 17383934

[61] Shechter A, Boivin DB. Sleep, Hormones, and Circadian Rhythms throughout the Menstrual Cycle in Healthy Women and Women with Premenstrual Dysphoric Disorder. Int J Endocrinol. 2010;2010:259345. doi: 10.1155/2010/259345 20145718 PMC2817387

[62] Roenneberg T. How can social jetlag affect health? Nature Reviews Endocrinology. 2023;19(7):383–4. doi: 10.1038/s41574-023-00851-2 PMC10204006 37221400

[63] Wittmann M, Dinich J, Merrow M, Roenneberg T. Social jetlag: misalignment of biological and social time. Chronobiol Int. 2006;23(1–2):497–509. doi: 10.1080/07420520500545979 16687322

[64] Rattenborg NC, de la Iglesia HO, Kempenaers B, Lesku JA, Meerlo P, Scriba MF. Sleep research goes wild: new methods and approaches to investigate the ecology, evolution and functions of sleep. Philos Trans R Soc Lond B Biol Sci. 2017;372(1734):20160251. doi: 10.1098/rstb.2016.0251 28993495 PMC5647278

[65] Yetish G, Kaplan H, Gurven M, Wood B, Pontzer H, Manger PR, et al. Natural sleep and its seasonal variations in three pre-industrial societies. Curr Biol. 2015;25(21):2862–8. doi: 10.1016/j.cub.2015.09.046 26480842 PMC4720388

[66] Skidmore PML, Howe AS, Polak MA, Wong JE, Lubransky A, Williams SM, et al. Sleep duration and adiposity in older adolescents from Otago, New Zealand: relationships differ between boys and girls and are independent of food choice. Nutr J. 2013;12:128. doi: 10.1186/1475-2891-12-128 24034352 PMC3848574

[67] Cheng SH, Shih C-C, Lee IH, Hou Y-W, Chen KC, Chen K-T, et al. A study on the sleep quality of incoming university students. Psychiatry Res. 2012;197(3):270–4. doi: 10.1016/j.psychres.2011.08.011 22342120

[68] Liu Y, Zhang J, Li SX, Chan NY, Yu MWM, Lam SP, et al. Excessive daytime sleepiness among children and adolescents: prevalence, correlates, and pubertal effects. Sleep Med. 2019;53:1–8. doi: 10.1016/j.sleep.2018.08.028 30384136

[69] Boerner KE, Keogh E, Inkster AM, Nahman-Averbuch H, Oberlander TF. A developmental framework for understanding the influence of sex and gender on health: Pediatric pain as an exemplar. Neurosci Biobehav Rev. 2024;158:105546. doi: 10.1016/j.neubiorev.2024.105546 38272336

[70] Brown AMC, Gervais NJ. Role of Ovarian Hormones in the Modulation of Sleep in Females Across the Adult Lifespan. Endocrinology. 2020;161(9):bqaa128. doi: 10.1210/endocr/bqaa128 32735650 PMC7450669

[71] Wu Y, Gong Q, Zou Z, Li H, Zhang X. Short sleep duration and obesity among children: A systematic review and meta-analysis of prospective studies. Obes Res Clin Pract. 2017;11(2):140–50. doi: 10.1016/j.orcp.2016.05.005 27269366

[72] Dorofaeff TF, Denny S. Sleep and adolescence. Do New Zealand teenagers get enough? J Paediatr Child Health. 2006;42(9):515–20. doi: 10.1111/j.1440-1754.2006.00914.x 16925537

[73] Godsell S, White J. Adolescent perceptions of sleep and influences on sleep behaviour: A qualitative study. J Adolesc. 2019;73:18–25. doi: 10.1016/j.adolescence.2019.03.010 30953841

[74] Li X, Li H, Luo J. Cross-lagged panel analysis of the relationship between social networking sites use (SNSU) and sleep problems among university students. BMC Public Health. 2024;24(1):2283. doi: 10.1186/s12889-024-19840-9 39174938 PMC11342664

[75] Madrid-Valero JJ, Kirkpatrick RM, González-Javier F, Gregory AM, Ordoñana JR. Sex differences in sleep quality and psychological distress: Insights from a middle-aged twin sample from Spain. J Sleep Res. 2023;32(2):e13714. doi: 10.1111/jsr.13714 36054078

[76] Chen TQ, Dong B, Zhang WJ, Gao DS, Dong YH, Ma J. Association between speed and endurance performance with sleep duration in children and adolescents. J Peking Univ Health Sci. 2018;50(3):429–35. 29930409

[77] Wang Y, Wang J, Wu H, Ma Y. A study of the relationship between physical fitness index and sleep quality in Chinese adolescents. Sleep Breath. 2025;29(6):372. doi: 10.1007/s11325-025-03541-7 41324782

[78] Min L, Wang D, You Y, Fu Y, Ma X. Effects of High-Intensity Interval Training on Sleep: A Systematic Review and Meta-Analysis. Int J Environ Res Public Health. 2021;18(20):10973. doi: 10.3390/ijerph182010973 34682718 PMC8535574

[79] Suppiah H, Chia M. The Somnolent Youth-Sleep and the Influence of Exercise: A Narrative Review. Sports. 2015;3(2):116–35. doi: 10.3390/sports3020116

[80] Sobral HD, Correia Júnior JL, Duda MBF, Gonçalves MP, Cardoso MD, Dias RDF. Perfil de aptidão física relacionada à saúde em adolescentes brasileiros: Revisão Integrativa da Literatura / Health-Related Fitness Profile in Brazilian Adolescents: An Integrative Literature Review. IDonline. 2022;16(61):228–36. doi: 10.14295/idonline.v16i61.3507

[81] Wijayasiri UKDC, Wimalasekere SW, Ishikawa Y, Waidyasekara H, Sivayogan S. Physiological Approach to Cardio-endurance Training: Indicators of Optimal Parasympathetic Input on Cardiovascular Regulation are Better Predictors of Running Performance of Distance Runners. EJSPORT. 2023;3(6):1–9. doi: 10.24018/ejsport.2023.3.6.97

[82] Sanz-Martín D, Ubago-Jiménez JL, Ruiz-Tendero G, Zurita-Ortega F, Melguizo-Ibáñez E, Puertas-Molero P. The Relationships between Physical Activity, Screen Time and Sleep Time According to the Adolescents’ Sex and the Day of the Week. Healthcare (Basel). 2022;10(10):1955. doi: 10.3390/healthcare10101955 36292402 PMC9601728

[83] Kruk J, Aboul-Enein BH, Duchnik E. Exercise-induced oxidative stress and melatonin supplementation: current evidence. J Physiol Sci. 2021;71(1):27. doi: 10.1186/s12576-021-00812-2 34470608 PMC8409271

[84] Brand S, Kalak N, Gerber M, Clough PJ, Lemola S, Sadeghi Bahmani D, et al. During early to mid adolescence, moderate to vigorous physical activity is associated with restoring sleep, psychological functioning, mental toughness and male gender. J Sports Sci. 2017;35(5):426–34. doi: 10.1080/02640414.2016.1167936 27033183

[85] Mitchell JA, Godbole S, Moran K, Murray K, James P, Laden F, et al. No Evidence of Reciprocal Associations between Daily Sleep and Physical Activity. Med Sci Sports Exerc. 2016;48(10):1950–6. doi: 10.1249/MSS.0000000000001000 27285490 PMC5026562

[86] Kong Z, Wei X, Shen M, Cheng Y, Feng J. Interval training has more negative effects on sleep in adolescent speed skaters: a randomized cross controlled trial. Front Sports Act Living. 2024;6:1367190. doi: 10.3389/fspor.2024.1367190 38689870 PMC11058656

[87] Cajochen C, Frey S, Anders D, Späti J, Bues M, Pross A, et al. Evening exposure to a light-emitting diodes (LED)-backlit computer screen affects circadian physiology and cognitive performance. J Appl Physiol (1985). 2011;110(5):1432–8. doi: 10.1152/japplphysiol.00165.2011 21415172

[88] Chang A-M, Aeschbach D, Duffy JF, Czeisler CA. Evening use of light-emitting eReaders negatively affects sleep, circadian timing, and next-morning alertness. Proc Natl Acad Sci U S A. 2015;112(4):1232–7. doi: 10.1073/pnas.1418490112 25535358 PMC4313820

[89] Windred DP, Burns AC, Lane JM, Olivier P, Rutter MK, Saxena R, et al. Brighter nights and darker days predict higher mortality risk: A prospective analysis of personal light exposure in >88,000 individuals. Proc Natl Acad Sci U S A. 2024;121(43):e2405924121. doi: 10.1073/pnas.2405924121 39405349 PMC11513964

[90] Arora T, Broglia E, Thomas GN, Taheri S. Associations between specific technologies and adolescent sleep quantity, sleep quality, and parasomnias. Sleep Med. 2014;15(2):240–7. doi: 10.1016/j.sleep.2013.08.799 24394730

[91] Del Rio João KA, de Jesus SN, Carmo C, Pinto P. Sleep quality components and mental health: Study with a non-clinical population. Psychiatry Res. 2018;269:244–50. doi: 10.1016/j.psychres.2018.08.020 30153603

[92] Ohayon MM, Roth T. What are the contributing factors for insomnia in the general population?. J Psychosom Res. 2001;51(6):745–55. doi: 10.1016/s0022-3999(01)00285-9 11750297

[93] Tsapanou A, Gu Y, O’Shea DM, Yannakoulia M, Kosmidis MH, Dardiotis E, et al. Dataset on the associations between sleep quality/duration and cognitive performance in cognitively healthy older adults. Data Brief. 2017;14:720–3. doi: 10.1016/j.dib.2017.08.028 28932777 PMC5596325

[94] Baldwin CM, Ervin A-M, Mays MZ, Robbins J, Shafazand S, Walsleben J, et al. Sleep disturbances, quality of life, and ethnicity: the Sleep Heart Health Study. J Clin Sleep Med. 2010;6(2):176–83. doi: 10.5664/jcsm.27768 20411696 PMC2854706

[95] Barclay NL, Eley TC, Maughan B, Rowe R, Gregory AM. Associations between diurnal preference, sleep quality and externalizing behaviours: a behavioural genetic analysis. Psychol Med. 2011;41(5):1029–40. doi: 10.1017/S0033291710001741 20836908

[96] Li Y, Guo K. Research on the relationship between physical activity, sleep quality, psychological resilience, and social adaptation among Chinese college students: A cross-sectional study. Front Psychol. 2023;14:1104897. doi: 10.3389/fpsyg.2023.1104897 36844303 PMC9950505

[97] Li G, Chen Y, Chaudhary S, Li CS, Hao D, Yang L, et al. Sleep dysfunction mediates the relationship between hypothalamic-insula connectivity and anxiety-depression symptom severity bidirectionally in young adults. Neuroimage. 2023;279:120340. doi: 10.1016/j.neuroimage.2023.120340 37611815

[98] Senior M, Williams H, Higgs G. Urban-rural mortality differentials: controlling for material deprivation. Soc Sci Med. 2000;51(2):289–305. doi: 10.1016/s0277-9536(99)00454-2 10832575

[99] Pampalon R, Hamel D, Gamache P. Health inequalities in urban and rural Canada: comparing inequalities in survival according to an individual and area-based deprivation index. Health Place. 2010;16(2):416–20. doi: 10.1016/j.healthplace.2009.11.012 20022551

[100] Guo L, Liu F, Zhu W, Cheng J, Zhang L, Zhang B, et al. Pathways from early-life urbanicity to adult neurobehavioral traits via menarche timing. Nat Cities. 2025;2(12):1226–39. doi: 10.1038/s44284-025-00352-5

[101] Gong P, Liang S, Carlton EJ, Jiang Q, Wu J, Wang L. Urbanisation and health in China. The Lancet. 2012;379(9818):843–52. doi: 10.1016/S0140-6736(11)61878-3 PMC3733467 22386037

[102] Wang L-B, Gong Y-C, Fang Q-L, Cui X-X, Dharmage SC, Jalaludin B, et al. Association Between Exposure to Outdoor Artificial Light at Night and Sleep Disorders Among Children in China. JAMA Netw Open. 2022;5(5):e2213247. doi: 10.1001/jamanetworkopen.2022.13247 35594042 PMC9123501

[103] Jiang S, Ren Q, Jiang C, Wang L. Academic stress and depression of Chinese adolescents in junior high schools: Moderated mediation model of school burnout and self-esteem. J Affect Disord. 2021;295:384–9. doi: 10.1016/j.jad.2021.08.085 34492431

[104] Xu X, Piao W, Fang H, Guo Q, Ju L, Cai S, et al. Beverage consumption of children and adolescents aged 6-17 years - China, 2016-2017. China CDC Wkly. 2021;3(13):279–84. doi: 10.46234/ccdcw2021.064 34594866 PMC8392978

[105] Garbers S, Suruki C, Falletta KA, Gold MA, Bruzzese J-M. Psychosocial stress, sleep quality and interest in mind-body integrative health sleep intervention among urban adolescents in the school-based health setting. Complement Ther Med. 2021;58:102714. doi: 10.1016/j.ctim.2021.102714 33766621 PMC10119780

[106] Zhang L, Chen J, Zhang J, Wu W, Huang K, Chen R, et al. Regional Disparities in Obesity Among a Heterogeneous Population of Chinese Children and Adolescents. JAMA Netw Open. 2021;4(10):e2131040. doi: 10.1001/jamanetworkopen.2021.31040 34698846 PMC8548942

[107] Sun Y, Zhong Y, Sun W, Chu L, Long J, Fan XW. More prevalent and more severe: gender differences of depressive symptoms in Chinese adolescents. Front Public Health. 2023;11:1167234. doi: 10.3389/fpubh.2023.1167234 37521991 PMC10372346

[108] Bao C, Han L. Gender difference in anxiety and related factors among adolescents. Front Public Health. 2025;12:1410086. doi: 10.3389/fpubh.2024.1410086 39830180 PMC11738925

[109] Guo B, Wu Q, Gong J, Xiao Z, Tang Y, Shang J, et al. Gender Difference in Body Fat for Healthy Chinese Children and Adolescents. Child Obes. 2016;12(2):144–54. doi: 10.1089/chi.2015.0139 26974254

[110] Mezzofranco L, Agostini L, Boutarbouche A, Melato S, Zalunardo F, Franco A, et al. Sleep Habits and Disorders in School-Aged Children: A Cross-Sectional Study Based on Parental Questionnaires. Children (Basel). 2025;12(4):489. doi: 10.3390/children12040489 40310152 PMC12025705

[111] Buysse DJ. Sleep health: can we define it? Does it matter? Sleep. 2014;37(1):9–17. doi: 10.5665/sleep.3298 24470692 PMC3902880

