# Supplementary material for: Gender and residential differences in sleep quality among Chinese adolescents aged 13–18 years
Source: PLoS One. 2026 Jun 10;21(6):e0349681. doi: 10.1371/journal.pone.0349681 (PMC13252746; doi:10.1371/journal.pone.0349681)
Supplement: S1 Appendix — (DOCX) [file pone.0349681.s001.docx]

**Appendix File.1.** Sleep Quality: single predictor logistic regression models for the possibility of reporting a “Poor” score on the PSQI (scores >5) and six of the sleep factor components (scores >1)

| Variable | PSQI Total | | Subjective sleep quality | | Sleep latency | | Sleep duration | | Sleep disturbance | | Sleep medications | | Daytime dysfunction | |
| --- | --- | --- | --- | --- | --- | --- | --- | --- | --- | --- | --- | --- | --- | --- |
|  | OR (95% CI) | *p* | OR (95% CI) | *p* | OR (95% CI) | *p* | OR (95% CI) | *p* | OR (95% CI) | *p* | OR (95% CI) | *p* | OR (95% CI) | *p* |
| Residence | 0.84 (0.75, 0.94) | **0.00** | 1.04 (0.90, 1.20) | 0.63 | 0.83 (0.73, 0.94) | **0.00** | 0.67 (0.57, 0.80) | **0.00** | 0.86 (0.71, 1.03) | 0.09 | 1.08 (0.74, 1.57) | 0.71 | 0.98 (0.88, 1.08) | 0.65 |
| Gender | 1.51 (1.35, 1.69) | **0.00** | 1.17 (1.01, 1.35) | **0.04** | 1.08 (0.95, 1.23) | 0.22 | 1.59 (1.34, 1.88) | **0.00** | 1.64 (1.36, 1.98) | **0.00** | 1.53 (1.05, 2.25) | **0.03** | 1.67 (1.50, 1.85) | **0.00** |
| BMI | 1.06 (1.04, 1.08) | **0.00** | 1.08 (1.06, 1.10) | **0.00** | 1.06 (1.04, 1.08) | **0.00** | 1.06 (1.03, 1.08) | **0.00** | 1.10 (1.08, 1.13) | **0.00** | 1.13 (1.07, 1.18) | **0.00** | 1.04 (1.02, 1.05) | **0.00** |
| PFI | 0.92 (0.87, 0.97) | **0.00** | 0.93 (0.86, 1.00) | 0.06 | 0.88 (0.82, 0.94) | **0.00** | 1.06 (0.97, 1.15) | 0.19 | 0.76 (0.68, 0.84) | **0.00** | 1.01 (0.83, 1.23) | 0.95 | 0.99 (0.93, 1.04) | 0.60 |
| MVPA | 1.00 (1.00, 1.00) | **0.03** | 1.00 (1.00, 1.00) | 0.55 | 1.00 (1.00, 1.00) | **0.03** | 1.00 (1.00, 1.00) | **0.00** | 1.00 (1.00, 1.00) | **0.02** | 1.00 (1.00, 1.00) | 0.51 | 1.00 (1.00, 1.00) | **0.00** |
| Sedentary time | 1.04 (1.03, 1.05) | **0.00** | 1.03 (1.01, 1.05) | **0.00** | 1.00 (0.99, 1.02) | 0.90 | 1.07 (1.04, 1.09) | **0.00** | 1.01 (0.99, 1.03) | 0.46 | 1.01 (0.97, 1.05) | 0.67 | 1.06 (1.05, 1.07) | **0.00** |
| Screen time | 1.16 (1.10, 1.22) | **0.00** | 1.16 (1.09, 1.24) | **0.00** | 1.13 (1.07, 1.20) | **0.00** | 0.86 (0.79, 0.93) | **0.00** | 1.19 (1.09, 1.29) | **0.00** | 1.16 (0.97, 1.38) | 0.11 | 1.16 (1.10, 1.22) | **0.00** |
| Mental health | 0.92 (0.92, 0.93) | **0.00** | 0.93 (0.92, 0.94) | **0.00** | 0.94 (0.94, 0.95) | **0.00** | 0.98 (0.97, 0.99) | **0.00** | 0.93 (0.92, 0.94) | **0.00** | 0.97 (0.95, 0.99) | **0.00** | 0.93 (0.92, 0.93) | **0.00** |
| BMI Quadratic | 1.00 (1.00, 1.00) | 0.13 | 1.00 (1.00, 1.00) | 0.72 | 1.00 (1.00, 1.00) | 0.87 | 1.00 (1.00, 1.00) | 0.83 | 1.00 (1.00, 1.00) | 0.28 | 1.00 (1.00, 1.00) | 0.31 | 1.00 (1.00, 1.00) | 0.66 |
| PFI Quadratic | 1.00 (0.99, 1.02) | 0.85 | 1.00 (0.99, 1.02) | 0.70 | 1.00 (0.99, 1.01) | 0.85 | 1.01 (0.99, 1.02) | 0.34 | 0.99 (0.97, 1.00) | 0.09 | 0.99 (0.91, 1.08) | 0.79 | 1.00 (0.98, 1.01) | 0.47 |
| MVPA Quadratic | 1.00 (1.00, 1.00) | 0.24 | 1.00 (1.00, 1.00) | 0.55 | 1.00 (1.00, 1.00) | 0.23 | 1.00 (1.00, 1.00) | **0.01** | 1.00 (1.00, 1.00) | 0.48 | 1.00 (1.00, 1.00) | 0.41 | 1.00 (1.00, 1.00) | **0.01** |
| Sitting time  Quadratic | 1.00 (1.00, 1.00) | 0.10 | 1.00 (1.00, 1.00) | 0.59 | 1.00 (1.00, 1.00) | 0.92 | 1.00 (1.00, 1.00) | 0.34 | 1.00 (1.00, 1.01) | **0.01** | 1.00 (1.00, 1.01) | 0.22 | 1.00 (0.99, 1.00) | **0.00** |
| Screen time  Quadratic | 0.99 (0.98, 1.00) | **0.00** | 0.99 (0.98, 1.00) | **0.04** | 0.99 (0.99, 1.00) | 0.11 | 1.02 (1.01, 1.03) | **0.00** | 0.99 (0.98, 1.00) | 0.07 | 0.98 (0.95, 1.01) | 0.29 | 0.99 (0.98, 0.99) | **0.00** |
| Mental health  Quadratic | 1.00 (1.00, 1.00) | **0.00** | 1.00 (1.00, 1.00) | **0.00** | 1.00 (1.00, 1.00) | **0.00** | 1.00 (1.00, 1.00) | 0.73 | 1.00 (1.00, 1.00) | **0.00** | 1.00 (1.00, 1.00) | 0.32 | 1.00 (1.00, 1.00) | **0.00** |

Significant values are highlighted in bold.

BMI, body mass index; PFI, Physical fitness index; MVPA, Moderate to vigorous physical activity; CI, confidence interval; OR, odds ratio; PSQI, Pittsburgh Sleep Quality Index.
