# Supplementary material for: Gender and residential differences in sleep quality among Chinese adolescents aged 13–18 years
Source: PLoS One. 2026 Jun 10;21(6):e0349681. doi: 10.1371/journal.pone.0349681 (PMC13252746; doi:10.1371/journal.pone.0349681)
Supplement: Attachment — Submitted filename: Response_to_Reviewers.docx [file pone.0349681.s004.docx]

**Response to Reviewers**

*Manuscript ID: PONE-D-26-07223*

Dear Dr. Tanja Grubić Kezele and Reviewers,

We sincerely thank you and the reviewers for the careful evaluation of our manuscript entitled “Gender and residential differences in sleep quality among Chinese adolescents aged 13–18 years” (Manuscript ID: PONE-D-26-07223). We are very grateful for the constructive comments and thoughtful suggestions, which have helped us improve the manuscript substantially.

We have carefully revised the manuscript in accordance with all comments raised by the Academic Editor and the reviewers. Below, we provide a detailed point-by-point response describing each modification made in the revised manuscript.

# Responses to the Academic Editor / Journal Requirements

**Comment:** Please ensure that the manuscript meets PLOS ONE style requirements, including file naming and manuscript formatting.

**Response:** We carefully revised the manuscript to improve conformity with PLOS ONE style requirements. Specifically, we reformatted the entire reference list according to the journal style, moved all tables from the end of the manuscript to the paragraphs immediately following their first citation in the main text, revised the section heading from “Materials and Methods” to “Materials and methods,” removed the “Authors’ information” section, and deleted the “Contributing authors” email addresses from the title page.

**Comment:** The ethics statement should appear only in the Methods section of the manuscript.

**Response:** We revised the manuscript accordingly. The ethics statement now appears only in the Materials and methods section. We deleted the ethics-related “Trial registration” statement from the Abstract and removed the ethics- and consent-related statements from the Declarations section.

**Comment:** Please clarify the data availability statement and the data-sharing plan.

**Response:** In response, all authors agreed to make the study data publicly available. We revised the “Availability of data and materials” statement to read: “All relevant data are within the manuscript and its Supporting Information files.” In addition, the anonymized raw dataset has been uploaded as S2 Appendix (Raw data).

**Comment:** Figure 1 may contain copyrighted map or satellite material and must be replaced with a figure that complies with CC BY 4.0 or accompanied by appropriate permission.

**Response:** We replaced the original Figure 1 with a newly prepared figure based on publicly available Natural Earth map data. To make the source explicit, we also added the caption text: “Base map of China data were obtained from Natural Earth.”

**Comment:** Please include captions for the Supporting Information files at the end of the manuscript and update the in-text citations accordingly.

**Response:** We revised the manuscript accordingly. After the reference list, we added a dedicated “Supporting information” section and included the following captions: “S1 Appendix. Sleep Quality: single predictor logistic regression models for the possibility of reporting a ‘Poor’ score on the PSQI (scores >5) and six of the sleep factor components (scores >1)” and “S2 Appendix. Raw data.” We also updated the corresponding in-text citation in the section “Correlates of poor sleep quality.”

**Comment:** The previous raw data file may contain identifying information. Please remove or anonymize all personal information and re-upload a fully anonymized dataset.

**Response:** We carefully reviewed the dataset and confirmed that the newly uploaded S2 Appendix contains no personally identifiable information. The dataset does not include names, initials, addresses, exact dates, contact information, IP addresses, location data, or specific identifying codes. Although sex is retained as a study variable, the participants were randomly selected school-aged children, and the shared dataset does not permit identification of individual participants. We therefore believe that the uploaded dataset is fully anonymized and consistent with participant privacy protection and consent requirements.

**Comment:** If reviewer comments include recommendations to cite specific previously published works, please evaluate whether these publications are relevant and should be cited.

**Response:** We carefully reviewed the literature suggested by Reviewer 2 and concluded that it is relevant to the present study. Accordingly, we added the recommended article by Mezzofranco et al. and one additional related article by Buysse to support the newly added integrated interpretation of multidimensional and clustered sleep problems in the Discussion section.

**Comment:** Please review the reference list to ensure that it is complete and correct and address any retracted, duplicated, or incorrect references.

**Response:** We carefully rechecked the entire reference list. We confirmed that the references cited in this study were not retracted at the time of revision. We also corrected the reference list by removing duplicated references, deleting an incorrectly cited reference, correcting an author-name error in the Discussion text, and converting the originally cited Chinese references into English citation format where appropriate.

# Response to Reviewer 1

**Comment:** Potential unmeasured factors such as pre-sleep tea/coffee intake, bedtime reading, electronic devices in the bedroom, and the presence of roommates or siblings may also affect sleep quality and should be discussed as limitations.

**Response:** We thank the reviewer for this valuable suggestion. In response, we added a new paragraph to the “Limitation of this study” section to acknowledge that screen time and sedentary time were assessed as total daily duration and that several sleep-hygiene and bedroom-environment factors were not specifically measured. The added text explicitly discusses pre-sleep caffeine intake, bedtime reading habits, the presence and use of electronic devices in the bedroom before sleep, and whether siblings or roommates were present during sleep as potentially relevant but unmeasured factors.

# Response to Reviewer 2

**Comment:** The cross-sectional design and reliance on self-reported measures should be more clearly acknowledged as limitations. A clearer statement of the study’s specific contribution would also strengthen the manuscript.

**Response:** We appreciate this helpful comment. We revised the “Limitation of this study” section to provide a clearer statement of the study’s specific contribution and a more explicit acknowledgment of the limitations associated with the cross-sectional design and the reliance on self-reported measures. We now clarify that the cross-sectional design does not allow causal inference or determination of directionality, and that the use of self-reported questionnaires may have introduced recall bias, reporting bias, and social desirability bias. We also added a statement explaining that the present study contributes by simultaneously examining sleep quality in relation to multiple physical- and mental-health-related factors in a large multi-city sample, while also evaluating the moderating roles of residence and gender.

**Comment:** Sleep and its disturbances may occur in clusters rather than as isolated conditions. A more integrated perspective could improve interpretation of the findings.

**Response:** Thank you for this insightful suggestion. We agreed that adolescent sleep problems are often interrelated and multidimensional rather than isolated conditions. Therefore, we added a new paragraph to the end of the Discussion section explaining that sleep quality problems in adolescents may exist in interrelated and clustered forms. In this paragraph, we cite Mezzofranco et al. and Buysse to support this integrated perspective and to help interpret why multiple PSQI dimensions showed unfavorable patterns within the same population.

# Additional Revisions Identified During the Authors’ Re-Examination

**Response:** In addition to the revisions made in direct response to the Academic Editor’s and reviewers’ comments, we also corrected several errors identified during our own re-examination of the manuscript. First, in the Statistical analyses section, we corrected the reversed classification of “poor” and “good” for PSQI component scores. Second, in the original Table 2, the percentages for male and female participants in the sleep-quality percentage rows had been inadvertently transposed; these values have now been corrected in the revised manuscript.

We once again sincerely thank the Academic Editor and the reviewers for their careful reading of our manuscript and for their constructive comments. We believe that the revisions have substantially improved the clarity, rigor, and presentation of the manuscript, and we respectfully submit the revised version for your further consideration.

Yours sincerely,

Xuye Kang

On behalf of all authors
