# Supplementary material for: Gender and residential differences in sleep quality among Chinese adolescents aged 13–18 years
Source: PLoS One. 2026 Jun 10;21(6):e0349681. doi: 10.1371/journal.pone.0349681 (PMC13252746; doi:10.1371/journal.pone.0349681)
Supplement: Peer review history

# Peer review: Decision Letter 0

**DOI:** 10.1371/journal.pone.0349681.r001

**Authors:** Tanja Grubić Kezele

14 Apr 2026

PONE-D-26-07223

Gender and residential differences in sleep quality among Chinese adolescents aged 13-18 years

PLOS One

Dear Dr. li,

Thank you for submitting your manuscript to PLOS ONE. After careful consideration, we feel that it has merit but does not fully meet PLOS ONE’s publication criteria as it currently stands. Therefore, we invite you to submit a revised version of the manuscript that addresses the points raised during the review process.

==============================

Based on the reviewers' suggestions, the paper needs a minor revision. The reviewers' comments can be found below.

==============================

Please submit your revised manuscript by May 29 2026 11:59PM. If you will need more time than this to complete your revisions, please reply to this message or contact the journal office at plosone@plos.org. When you're ready to submit your revision, log on to https://www.editorialmanager.com/pone/ and select the 'Submissions Needing Revision' folder to locate your manuscript file.

Please include the following items when submitting your revised manuscript:

- A letter that responds to each point raised by the academic editor and reviewer(s). You should upload this letter as a separate file labeled 'Response to Reviewers'.
- A marked-up copy of your manuscript that highlights changes made to the original version. You should upload this as a separate file labeled 'Revised Manuscript with Track Changes'.
- An unmarked version of your revised paper without tracked changes. You should upload this as a separate file labeled 'Manuscript'.

If you would like to make changes to your financial disclosure, please include your updated statement in your cover letter. Guidelines for resubmitting your figure files are available below the reviewer comments at the end of this letter.

If applicable, we recommend that you deposit your laboratory protocols in protocols.io to enhance the reproducibility of your results. Protocols.io assigns your protocol its own identifier (DOI) so that it can be cited independently in the future. For instructions see: https://journals.plos.org/plosone/s/submission-guidelines#loc-laboratory-protocols. Additionally, PLOS ONE offers an option for publishing peer-reviewed Lab Protocol articles, which describe protocols hosted on protocols.io. Read more information on sharing protocols at https://plos.org/protocols?utm_medium=editorial-email&utm_source=authorletters&utm_campaign=protocols.

As the corresponding author, your ORCID iD is verified in the submission system and will appear in the published article. PLOS supports the use of ORCID, and we encourage all coauthors to register for an ORCID iD and use it as well. Please encourage your coauthors to verify their ORCID iD within the submission system before final acceptance, as unverified ORCID iDs will not appear in the published article. *Only* the individual author can complete the verification step; PLOS staff *cannot* verify ORCID iDs on behalf of authors.

We look forward to receiving your revised manuscript.

Kind regards,

Tanja Grubić Kezele, Ph.D., M.D.

Academic Editor

PLOS One

Journal Requirements:

When submitting your revision, we need you to address these additional requirements.

1. Please ensure that your manuscript meets PLOS ONE's style requirements, including those for file naming. The PLOS ONE style templates can be found at

https://journals.plos.org/plosone/s/file?id=wjVg/PLOSOne_formatting_sample_main_body.pdf and https://journals.plos.org/plosone/s/file?id=ba62/PLOSOne_formatting_sample_title_authors_affiliations.pdf

2. Your ethics statement should only appear in the Methods section of your manuscript. If your ethics statement is written in any section besides the Methods, please delete it from any other section.

3. When completing the data availability statement of the submission form, you indicated that you will make your data available on acceptance. We strongly recommend all authors decide on a data sharing plan before acceptance, as the process can be lengthy and hold up publication timelines. Please note that, though access restrictions are acceptable now, your entire data will need to be made freely accessible if your manuscript is accepted for publication. This policy applies to all data except where public deposition would breach compliance with the protocol approved by your research ethics board. If you are unable to adhere to our open data policy, please kindly revise your statement to explain your reasoning and we will seek the editor's input on an exemption. Please be assured that, once you have provided your new statement, the assessment of your exemption will not hold up the peer review process.

4. We note that Figure 1 in your submission contain [map/satellite] images which may be copyrighted. All PLOS content is published under the Creative Commons Attribution License (CC BY 4.0), which means that the manuscript, images, and Supporting Information files will be freely available online, and any third party is permitted to access, download, copy, distribute, and use these materials in any way, even commercially, with proper attribution. For these reasons, we cannot publish previously copyrighted maps or satellite images created using proprietary data, such as Google software (Google Maps, Street View, and Earth). For more information, see our copyright guidelines: http://journals.plos.org/plosone/s/licenses-and-copyright.

We require you to either (1) present written permission from the copyright holder to publish these figures specifically under the CC BY 4.0 license, or (2) remove the figures from your submission:

a. You may seek permission from the original copyright holder of Figure 1 to publish the content specifically under the CC BY 4.0 license.

We recommend that you contact the original copyright holder with the Content Permission Form (http://journals.plos.org/plosone/s/file?id=7c09/content-permission-form.pdf) and the following text:

"I request permission for the open-access journal PLOS ONE to publish XXX under the Creative Commons Attribution License (CCAL) CC BY 4.0 (http://creativecommons.org/licenses/by/4.0/). Please be aware that this license allows unrestricted use and distribution, even commercially, by third parties. Please reply and provide explicit written permission to publish XXX under a CC BY license and complete the attached form."

Please upload the completed Content Permission Form or other proof of granted permissions as an "Other" file with your submission.

In the figure caption of the copyrighted figure, please include the following text: “Reprinted from [ref] under a CC BY license, with permission from [name of publisher], original copyright [original copyright year].”

b. If you are unable to obtain permission from the original copyright holder to publish these figures under the CC BY 4.0 license or if the copyright holder’s requirements are incompatible with the CC BY 4.0 license, please either i) remove the figure or ii) supply a replacement figure that complies with the CC BY 4.0 license. Please check copyright information on all replacement figures and update the figure caption with source information. If applicable, please specify in the figure caption text when a figure is similar but not identical to the original image and is therefore for illustrative purposes only.

The following resources for replacing copyrighted map figures may be helpful:

USGS National Map Viewer (public domain): http://viewer.nationalmap.gov/viewer/

The Gateway to Astronaut Photography of Earth (public domain): http://eol.jsc.nasa.gov/sseop/clickmap/

Maps at the CIA (public domain): https://www.cia.gov/library/publications/the-world-factbook/index.html and https://www.cia.gov/library/publications/cia-maps-publications/index.html

NASA Earth Observatory (public domain): http://earthobservatory.nasa.gov/

Landsat: http://landsat.visibleearth.nasa.gov/

USGS EROS (Earth Resources Observatory and Science (EROS) Center) (public domain): http://eros.usgs.gov/#

Natural Earth (public domain): http://www.naturalearthdata.com/

5. Please include captions for your Supporting Information files at the end of your manuscript, and update any in-text citations to match accordingly. Please see our Supporting Information guidelines for more information: http://journals.plos.org/plosone/s/supporting-information.

6. We note that there is identifying data in the Supporting Information file < Raw data.xlsx >. Due to the inclusion of these potentially identifying data, we have removed this file from your file inventory. Prior to sharing human research participant data, authors should consult with an ethics committee to ensure data are shared in accordance with participant consent and all applicable local laws.

Data sharing should never compromise participant privacy. It is therefore not appropriate to publicly share personally identifiable data on human research participants. The following are examples of data that should not be shared:

-Name, initials, physical address

-Ages more specific than whole numbers

-Internet protocol (IP) address

-Specific dates (birth dates, death dates, examination dates, etc.)

-Contact information such as phone number or email address

-Location data

-ID numbers that seem specific (long numbers, include initials, titled “Hospital ID”) rather than random (small numbers in numerical order)

Data that are not directly identifying may also be inappropriate to share, as in combination they can become identifying. For example, data collected from a small group of participants, vulnerable populations, or private groups should not be shared if they involve indirect identifiers (such as sex, ethnicity, location, etc.) that may risk the identification of study participants.

Additional guidance on preparing raw data for publication can be found in our Data Policy (https://journals.plos.org/plosone/s/data-availability#loc-human-research-participant-data-and-other-sensitive-data) and in the following article: http://www.bmj.com/content/340/bmj.c181.long.

Please remove or anonymize all personal information (<specific identifying information in file to be removed>), ensure that the data shared are in accordance with participant consent, and re-upload a fully anonymized data set. Please note that spreadsheet columns with personal information must be removed and not hidden as all hidden columns will appear in the published file.

7. If the reviewer comments include a recommendation to cite specific previously published works, please review and evaluate these publications to determine whether they are relevant and should be cited. There is no requirement to cite these works unless the editor has indicated otherwise.

8. Please review your reference list to ensure that it is complete and correct. If you have cited papers that have been retracted, please include the rationale for doing so in the manuscript text, or remove these references and replace them with relevant current references. Any changes to the reference list should be mentioned in the rebuttal letter that accompanies your revised manuscript. If you need to cite a retracted article, indicate the article’s retracted status in the References list and also include a citation and full reference for the retraction notice.

[Note: HTML markup is below. Please do not edit.]

Reviewers' comments:

Reviewer's Responses to Questions

**Comments to the Author**

1. Is the manuscript technically sound, and do the data support the conclusions?

The manuscript must describe a technically sound piece of scientific research with data that supports the conclusions. Experiments must have been conducted rigorously, with appropriate controls, replication, and sample sizes. The conclusions must be drawn appropriately based on the data presented.

Reviewer #1: Yes

Reviewer #2: Yes

**********

2. Has the statistical analysis been performed appropriately and rigorously?

Reviewer #1: Yes

Reviewer #2: Yes

**********

3. Have the authors made all data underlying the findings in their manuscript fully available?

The PLOS Data policy requires authors to make all data underlying the findings described in their manuscript fully available without restriction, with rare exception (please refer to the Data Availability Statement in the manuscript PDF file). The data should be provided as part of the manuscript or its supporting information, or deposited to a public repository. For example, in addition to summary statistics, the data points behind means, medians and variance measures should be available. If there are restrictions on publicly sharing data—e.g. participant privacy or use of data from a third party—those must be specified.

Reviewer #1: Yes

Reviewer #2: Yes

**********

4. Is the manuscript presented in an intelligible fashion and written in standard English?

PLOS ONE does not copyedit accepted manuscripts, so the language in submitted articles must be clear, correct, and unambiguous. Any typographical or grammatical errors should be corrected at revision, so please note any specific errors here.

Reviewer #1: Yes

Reviewer #2: Yes

**********

5. Review Comments to the Author

Please use the space provided to explain your answers to the questions above. You may also include additional comments for the author, including concerns about dual publication, research ethics, or publication ethics. (Please upload your review as an attachment if it exceeds 20,000 characters)

Reviewer #1: Abstract fully describe the article.

The study's sample size is sufficient, supporting the reliability of the findings.

The purpose of the research is stated well.

The study is meticulously planned and maintained, presented in detail and systematically. Ethical rules are followed and the number of working groups is more than sufficient.The duration of the research is sufficient to reach a conclusion.The results have been sufficiently discussed in the literature and sufficient and appropriate literature is provided.

I just want to add a point that; Factors that could be ruled out include tea/coffee consumption before bedtime, reading a book, the presence of electronic devices (computer, television, cell phone) in the bedroom while sleeping, and the presence of a roommate/siblings. Since then, these issues can be discussed in the discussion as limitations.

Reviewer #2: This manuscript addresses an important public health topic and benefits from a large sample size and the inclusion of relevant factors such as gender, residence, physical activity, sedentary behavior, and mental health.

The findings are consistent with existing literature and provide a useful descriptive contribution. However, the cross-sectional design and reliance on self-reported measures should be more clearly acknowledged as limitations. A clearer statement of the study’s specific contribution would also strengthen the manuscript.

It may also be helpful to consider that sleep and its disturbances often occur in clusters rather than as isolated conditions. A more integrated perspective could enhance the interpretation of the results (see, for example, doi: 10.3390/children12040489; PMID: 40310152; PMCID: PMC12025705).

Overall, the manuscript is well organized and suitable for publication with minor revisions.

**********

6. PLOS authors have the option to publish the peer review history of their article (what does this mean?). If published, this will include your full peer review and any attached files.

If you choose “no”, your identity will remain anonymous but your review may still be made public.

**Do you want your identity to be public for this peer review?** For information about this choice, including consent withdrawal, please see our Privacy Policy.

Reviewer #1: **Yes:** Nurdan Tekgul

Reviewer #2: **Yes:** Luca Mezzofranco

**********

[NOTE: If reviewer comments were submitted as an attachment file, they will be attached to this email and accessible via the submission site. Please log into your account, locate the manuscript record, and check for the action link "View Attachments". If this link does not appear, there are no attachment files.]

To ensure your figures meet our technical requirements, please review our figure guidelines: https://journals.plos.org/plosone/s/figures

You may also use PLOS’s free figure tool, NAAS, to help you prepare publication quality figures: https://journals.plos.org/plosone/s/figures#loc-tools-for-figure-preparation.

NAAS will assess whether your figures meet our technical requirements by comparing each figure against our figure specifications.

# Author comment: Author response to Decision Letter 1

**DOI:** 10.1371/journal.pone.0349681.r002

22 Apr 2026

Dear Dr. Tanja Grubić Kezele and Reviewers,

We sincerely thank you and the reviewers for the careful evaluation of our manuscript entitled “Gender and residential differences in sleep quality among Chinese adolescents aged 13–18 years” (Manuscript ID: PONE-D-26-07223). We are very grateful for the constructive comments and thoughtful suggestions, which have helped us improve the manuscript substantially.

We have carefully revised the manuscript in accordance with all comments raised by the Academic Editor and the reviewers. Below, we provide a detailed point-by-point response describing each modification made in the revised manuscript.

Responses to the Academic Editor / Journal Requirements

Comment: Please ensure that the manuscript meets PLOS ONE style requirements, including file naming and manuscript formatting.

Response: We carefully revised the manuscript to improve conformity with PLOS ONE style requirements. Specifically, we reformatted the entire reference list according to the journal style, moved all tables from the end of the manuscript to the paragraphs immediately following their first citation in the main text, revised the section heading from “Materials and Methods” to “Materials and methods,” removed the “Authors’ information” section, and deleted the “Contributing authors” email addresses from the title page.

Comment: The ethics statement should appear only in the Methods section of the manuscript.

Response: We revised the manuscript accordingly. The ethics statement now appears only in the Materials and methods section. We deleted the ethics-related “Trial registration” statement from the Abstract and removed the ethics- and consent-related statements from the Declarations section.

Comment: Please clarify the data availability statement and the data-sharing plan.

Response: In response, all authors agreed to make the study data publicly available. We revised the “Availability of data and materials” statement to read: “All relevant data are within the manuscript and its Supporting Information files.” In addition, the anonymized raw dataset has been uploaded as S2 Appendix (Raw data).

Comment: Figure 1 may contain copyrighted map or satellite material and must be replaced with a figure that complies with CC BY 4.0 or accompanied by appropriate permission.

Response: We replaced the original Figure 1 with a newly prepared figure based on publicly available Natural Earth map data. To make the source explicit, we also added the caption text: “Base map of China data were obtained from Natural Earth.”

Comment: Please include captions for the Supporting Information files at the end of the manuscript and update the in-text citations accordingly.

Response: We revised the manuscript accordingly. After the reference list, we added a dedicated “Supporting information” section and included the following captions: “S1 Appendix. Sleep Quality: single predictor logistic regression models for the possibility of reporting a ‘Poor’ score on the PSQI (scores >5) and six of the sleep factor components (scores >1)” and “S2 Appendix. Raw data.” We also updated the corresponding in-text citation in the section “Correlates of poor sleep quality.”

Comment: The previous raw data file may contain identifying information. Please remove or anonymize all personal information and re-upload a fully anonymized dataset.

Response: We carefully reviewed the dataset and confirmed that the newly uploaded S2 Appendix contains no personally identifiable information. The dataset does not include names, initials, addresses, exact dates, contact information, IP addresses, location data, or specific identifying codes. Although sex is retained as a study variable, the participants were randomly selected school-aged children, and the shared dataset does not permit identification of individual participants. We therefore believe that the uploaded dataset is fully anonymized and consistent with participant privacy protection and consent requirements.

Comment: If reviewer comments include recommendations to cite specific previously published works, please evaluate whether these publications are relevant and should be cited.

Response: We carefully reviewed the literature suggested by Reviewer 2 and concluded that it is relevant to the present study. Accordingly, we added the recommended article by Mezzofranco et al. and one additional related article by Buysse to support the newly added integrated interpretation of multidimensional and clustered sleep problems in the Discussion section.

Comment: Please review the reference list to ensure that it is complete and correct and address any retracted, duplicated, or incorrect references.

Response: We carefully rechecked the entire reference list. We confirmed that the references cited in this study were not retracted at the time of revision. We also corrected the reference list by removing duplicated references, deleting an incorrectly cited reference, correcting an author-name error in the Discussion text, and converting the originally cited Chinese references into English citation format where appropriate.

Response to Reviewer 1

Comment: Potential unmeasured factors such as pre-sleep tea/coffee intake, bedtime reading, electronic devices in the bedroom, and the presence of roommates or siblings may also affect sleep quality and should be discussed as limitations.

Response: We thank the reviewer for this valuable suggestion. In response, we added a new paragraph to the “Limitation of this study” section to acknowledge that screen time and sedentary time were assessed as total daily duration and that several sleep-hygiene and bedroom-environment factors were not specifically measured. The added text explicitly discusses pre-sleep caffeine intake, bedtime reading habits, the presence and use of electronic devices in the bedroom before sleep, and whether siblings or roommates were present during sleep as potentially relevant but unmeasured factors.

Response to Reviewer 2

Comment: The cross-sectional design and reliance on self-reported measures should be more clearly acknowledged as limitations. A clearer statement of the study’s specific contribution would also strengthen the manuscript.

Response: We appreciate this helpful comment. We revised the “Limitation of this study” section to provide a clearer statement of the study’s specific contribution and a more explicit acknowledgment of the limitations associated with the cross-sectional design and the reliance on self-reported measures. We now clarify that the cross-sectional design does not allow causal inference or determination of directionality, and that the use of self-reported questionnaires may have introduced recall bias, reporting bias, and social desirability bias. We also added a statement explaining that the present study contributes by simultaneously examining sleep quality in relation to multiple physical- and mental-health-related factors in a large multi-city sample, while also evaluating the moderating roles of residence and gender.

Comment: Sleep and its disturbances may occur in clusters rather than as isolated conditions. A more integrated perspective could improve interpretation of the findings.

Response: Thank you for this insightful suggestion. We agreed that adolescent sleep problems are often interrelated and multidimensional rather than isolated conditions. Therefore, we added a new paragraph to the end of the Discussion section explaining that sleep quality problems in adolescents may exist in interrelated and clustered forms. In this paragraph, we cite Mezzofranco et al. and Buysse to support this integrated perspective and to help interpret why multiple PSQI dimensions showed unfavorable patterns within the same population.

Additional Revisions Identified During the Authors’ Re-Examination

Response: In addition to the revisions made in direct response to the Academic Editor’s and reviewers’ comments, we also corrected several errors identified during our own re-examination of the manuscript. First, in the Statistical analyses section, we corrected the reversed classification of “poor” and “good” for PSQI component scores. Second, in the original Table 2, the percentages for male and female participants in the sleep-quality percentage rows had been inadvertently transposed; these values have now been corrected in the revised manuscript.

We once again sincerely thank the Academic Editor and the reviewers for their careful reading of our manuscript and for their constructive comments. We believe that the revisions have substantially improved the clarity, rigor, and presentation of the manuscript, and we respectfully submit the revised version for your further consideration.

Yours sincerely,

Xuye Kang

On behalf of all authors

Attachment Submitted filename: Response_to_Reviewers.docx

# Editor report: Decision Letter 1

**DOI:** 10.1371/journal.pone.0349681.r003

**Authors:** Tanja Grubić Kezele

5 May 2026

Gender and residential differences in sleep quality among Chinese adolescents aged 13-18 years

PONE-D-26-07223R1

Dear Dr. li,

We’re pleased to inform you that your manuscript has been judged scientifically suitable for publication and will be formally accepted for publication once it meets all outstanding technical requirements.

Within one week, you’ll receive an e-mail detailing the required amendments. When these have been addressed, you’ll receive a formal acceptance letter and your manuscript will be scheduled for publication.

An invoice will be generated when your article is formally accepted. Please note, if your institution has a publishing partnership with PLOS and your article meets the relevant criteria, all or part of your publication costs will be covered. Please make sure your user information is up-to-date by logging into Editorial Manager at Editorial Manager® and clicking the ‘Update My Information' link at the top of the page. For questions related to billing, please contact billing support.

If your institution or institutions have a press office, please notify them about your upcoming paper to help maximize its impact. If they’ll be preparing press materials, please inform our press team as soon as possible -- no later than 48 hours after receiving the formal acceptance. Your manuscript will remain under strict press embargo until 2 pm Eastern Time on the date of publication. For more information, please contact onepress@plos.org.

Kind regards,

Tanja Grubić Kezele, Ph.D., M.D.

Academic Editor

PLOS One

Additional Editor Comments (optional):

Reviewers' comments:

# Editor report: Acceptance letter

**DOI:** 10.1371/journal.pone.0349681.r004

**Authors:** Tanja Grubić Kezele

PONE-D-26-07223R1

PLOS One

Dear Dr. li,

I'm pleased to inform you that your manuscript has been deemed suitable for publication in PLOS One. Congratulations! Your manuscript is now being handed over to our production team.

At this stage, our production department will prepare your paper for publication. This includes ensuring the following:

* All references, tables, and figures are properly cited

* All relevant supporting information is included in the manuscript submission,

* There are no issues that prevent the paper from being properly typeset

You will receive further instructions from the production team, including instructions on how to review your proof when it is ready. Please keep in mind that we are working through a large volume of accepted articles, so please give us a few days to review your paper and let you know the next and final steps.

Lastly, if your institution or institutions have a press office, please let them know about your upcoming paper now to help maximize its impact. If they'll be preparing press materials, please inform our press team within the next 48 hours. Your manuscript will remain under strict press embargo until 2 pm Eastern Time on the date of publication. For more information, please contact onepress@plos.org.

You will receive an invoice from PLOS for your publication fee after your manuscript has reached the completed accept phase. If you receive an email requesting payment before acceptance or for any other service, this may be a phishing scheme. Learn how to identify phishing emails and protect your accounts at https://explore.plos.org/phishing.

If we can help with anything else, please email us at customercare@plos.org.

Thank you for submitting your work to PLOS ONE and supporting open access.

Kind regards,

PLOS ONE Editorial Office Staff

on behalf of

Prof. dr. Tanja Grubić Kezele

Academic Editor

PLOS One
